# The embryonic origins of site-specific arthritis

**DOI:** 10.1038/s41590-026-02542-2

**Published:** 2026-06-08

**Authors:** Sarah Davidson, Davide Simone, Kathrin Jansen, Max Cowan, Caio Machado, Ian Reekie, Ananya Bhalla, Rowie Borst, Cesar Prada Medina, Joshua Bull, Zhi Yi Wong, Sarah Hill, Micon Garvilles, Sam Pledger, Patricia Reis Nisa, Nora Rebecca Schwingen, Dylan Windell, Moustafa Attar, Catherine Disney, Andrew J. Bodey, Alissa Parmenter, Helen Byrne, Sharif Ahmed, Shashidhara Marathe, Peter D. Lee, Chris Mahony, Adam P. Croft, Stephen Sansom, Mark C. Coles, Christopher D. Buckley

**Affiliations:** 1 Kennedy Institute of Rheumatology, University of Oxford, Oxford, UK; 2 Mathematical Institute, University of Oxford, Oxford, UK; 3 Diamond Light Source, Harwell Science and Innovation Campus, Oxford, UK; 4 Department of Mechanical Engineering, University College London, London, UK; 5 Rheumatology Research Group, University of Birmingham, Birmingham, UK; 6 Ludwig Institute for Cancer Research, University of Oxford, Oxford, UK

**Keywords:** Immunology, Cell biology

## Abstract

The cellular basis for site-specific inflammation remains unclear. In human fingers, proximal interphalangeal (PIP) joints are preferentially affected by inflammatory arthritis, whereas distal interphalangeal joints are spared, providing a model to investigate the predilection of inflammation to distinct sites. Here we combine single-cell RNA sequencing, imaging and X-ray tomography to examine cellular composition, spatial organization and structure of finger joints during fetal development. PIP joints had a larger synovial volume and were enriched for PI16^+^ ‘universal’ fibroblasts. These cells were located in perivascular regions and at developing tendon–ligament interfaces. PI16^+^ fibroblasts exhibited both a shared inflammatory and cell-type-specific response to cytokine stimulation, suggesting that the combination of their spatial location and transcriptional responses promote inflammation. We suggest that differences in the stoichiometry of mesenchymal cells established in utero, including the key role of PI16^+^ fibroblasts, is a general principle that drives inflammation susceptibility across tissues.

## Abstract (web summary)

Davidson et al. map the cellular and structural composition of developing joints and identify fibroblast populations that may predispose joints to inflammatory arthritis.

## Main

Rheumatoid arthritis (RA) is an immune-mediated inflammatory disease characterized by inflammation and damage of synovial joints. However, despite systemic immune activation, selective joint involvement is a hallmark of RA. For example, in fingers, the proximal interphalangeal (PIP) joint is typically inflamed, whereas the distal interphalangeal (DIP) joint is spared. Conversely, both joints can develop osteoarthritis (OA), a noninflammatory mechanical disease. The underlying causes of these arthritis tropisms remain an enigma.

Synovial fibroblasts play a critical role in RA pathology, comprising spatially and functionally distinct subsets that drive inflammation and joint damage^1–6^. Fibroblasts in the synovial lining layer (LL), a membrane-like structure that faces the joint cavity, promote cartilage and bone degradation, whereas sublining (SL) fibroblasts promote immune infiltration^1,6^. Although the ‘destructive’ LL is expanded in OA, ‘inflammatory’ SL fibroblasts dominate in RA, suggesting a relationship between the ratio of local stromal cells and arthritis pathology^2^.

Despite both functioning as hinge joints, the DIP and PIP joints have different mechanical properties and ranges of motion, with the PIP joint demonstrating greater flexibility enabling opposition to the thumb^7^. To investigate this specialization at a cellular and structural level, we examined the formation of human DIP and PIP joints during fetal development. The smaller scale of fetal joints provides a unique opportunity to examine the cellular and structural composition of whole joints without the need to biopsy different components.

Mouse models have shown that multiple progenitor populations give rise to different synovial joint structures. Joint development begins with formation of the interzone, a pool of GDF5^+^ mesenchymal-like progenitors. GDF5^+^ cells migrate out of the interzone and differentiate, giving rise to surrounding joint tissues including synovium, cartilage and ligaments^8^. Other joint structures, such as tendons, arise from GDF5^−^ progenitors, including scleraxis^+^ cells from peripheral regions of the developing joint. Although LL and SL fibroblasts have been identified in developing mouse knees, changes in stromal composition across different joints remain uninvestigated.

Using single-cell RNA sequencing (scRNAseq), a bespoke image analysis tool, high-resolution X-ray tomography and in vitro models, we highlight key differences in the cellular stoichiometry and structural architecture of developing finger joints. These data suggest that the functional specialization of these joints may ultimately dictate arthritis tropism.

## Results

### scRNAseq reveals a predominance of stromal cell precursors in developing finger joints

**Fig. 1:**
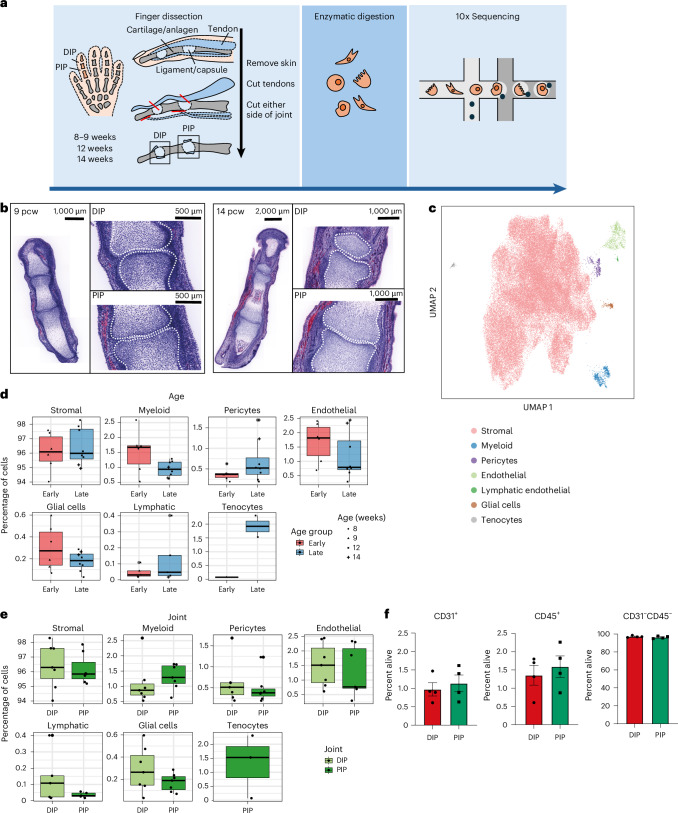
A single-cell atlas of fetal finger joints demonstrates that stromal cells are the dominant population between 8 and 14 pcw. **a**, Schematic depicting sample processing. DIP and PIP joints were dissected from 8–9 pcw fingers (early) and 12–14 pcw fingers (late), enzymatically digested to a single-cell suspension and run on the 10x Genomics platform. **b**, GE cellDIVE generated virtual hematoxylin and eosin (H&E) images of 9 and 14 pcw fingers, based on a background DAPI stain. White dotted lines indicate developing cartilage. **c**, Uniform manifold approximation and projection (UMAP) depicting major cell types detected in both DIP and PIP joints across all time points. **d**,**e**, Box plots showing the percentage of cell types, within each donor, across each time point (**d**) and joint type (**e**) at early (8–9 pcw) and late (12–14 pcw) stages from both PIP and DIP joints (early: *n* = 3, late: *n* = 4 donors). The center lines represent the median, the boxes show the interquartile range, and the whiskers extend to highest and lowest values within 1.5× the interquartile range. Data points outside the whiskers are plotted individually as outliers. Symbols represent each donor and indicate the sample age. **f**, Flow cytometry data from 14–15 pcw DIP and PIP joints showing the mean percentage of alive cells (*n* = 4 donors); error bars represent s.e.m.

**Extended Data Fig. 1:**
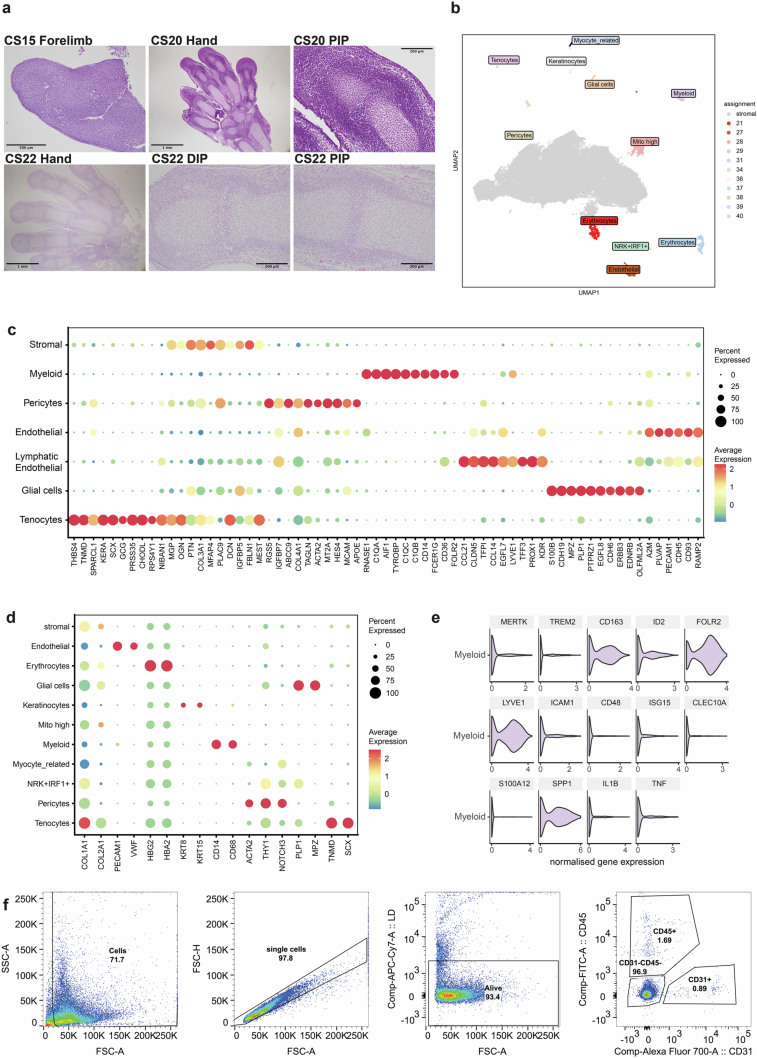
Histology depicting early joint development and marker expression across all cell types from the single cell atlas of foetal joints. H&E images of developing finger joints at different stages of development. (**a**) CS15 (top left), CS20 (top middle), ROI of PIP (top right), CS22 hand (bottom left), ROI DIP (bottom middle) and ROI PIP (bottom right). Images representative of n = 1. (**b**) UMAP depicting all cell types found to be present in the tissue samples. (**c**) Dotplot showing expression of top 10 markers for each cell type. (**d**) Dotplot showing expression of markers used to identify cell types. (**e**) Violin plots showing expression of markers, associated with resolving or inflammatory macrophages, in the embryonic myeloid cluster (defined in a). (**f**) The gating strategy employed to identify CD45-CD31- cells in 14-15pcw DIP and PIP joints.

To chart the cellular composition of developing finger joints, we generated an scRNAseq atlas of human fetal DIP and PIP joints. Skin was removed from fingers, and tendons were cut flush to the developing anlagen (Fig. 1a). Joints were excised by cutting either side of the joint space, leaving the cartilage and the surrounding joint soft tissue, such as ligaments, capsule and synovium. Single cells were isolated and sequenced from DIP and PIP joints from early (8–9 postconception weeks (pcw), *n* = 3) and late (12–14 pcw, *n* = 4) developmental stages. This captured early synovial development following cavitation, as well as more mature joint structures (Fig. 1b and Extended Data Fig. 1a). After quality control, the resulting dataset comprised 65,705 cells. Clustering and annotation revealed a small number of contaminating cells, which were excluded from further analysis (Extended Data Fig. 1b–d).

Of the remaining cells, 96.5% were stromal, comprising *COL1A1*^+^ early fibroblasts and *COL2A1*^+^ chondrocytes (Fig.1c and Extended Data Fig. 1d). Blood endothelial cells (*PECAM1*^+^*VWF*^+^; 1.42%), lymphatic endothelial cells (*LYVE1*^+^*PROX1*^+^; 0.059%), myeloid cells (*CD68*^+^*CD14*^+^; 1.05%), pericytes (*THY1*^+^*ACTA2*^+^*NOTCH3*^+^; 0.57%), glial cells (*PLP1*^+^*MPZ*^+^; 0.26%) and a few remaining tenocytes (*TNMD*^+^*SCX*^+^; 0.18%) were also identified (Fig. 1c–e and Extended Data Fig. 1c–e). Similar frequencies of these major cell types were found across time points and joint sites (Fig. 1d,e). Of note, myeloid cells expressed markers characteristic of a proresolving phenotype, including *CD163*, *ID2*, *FOLR2*, *LYVE1*, *MERTK* and *TREM2* (ref. ^9^), and exhibited low expression of genes encoding proinflammatory markers and cytokines, including *CD48*, *CLEC10A*, *S100A12*, *TNF* and *IL1B* (Extended Data Fig. 1e).

Flow cytometry validated the frequencies of endothelial (CD31^+^, 1.05%), immune (CD45^+^, 1.4%) and stromal cells (CD31^−^CD45^−^, 96.5%) at 14–15 pcw (Fig. 1f and Extended Data Fig. 1f). These data show that developing finger joints largely comprise *COL1A1*^+^*COL2A1*^+^ stromal cells and provide a resource to explore the developing cellular landscape of two distinct human finger joints.

### Stromal cells occupy distinct spatial niches within the developing finger joints

**Fig. 2:**
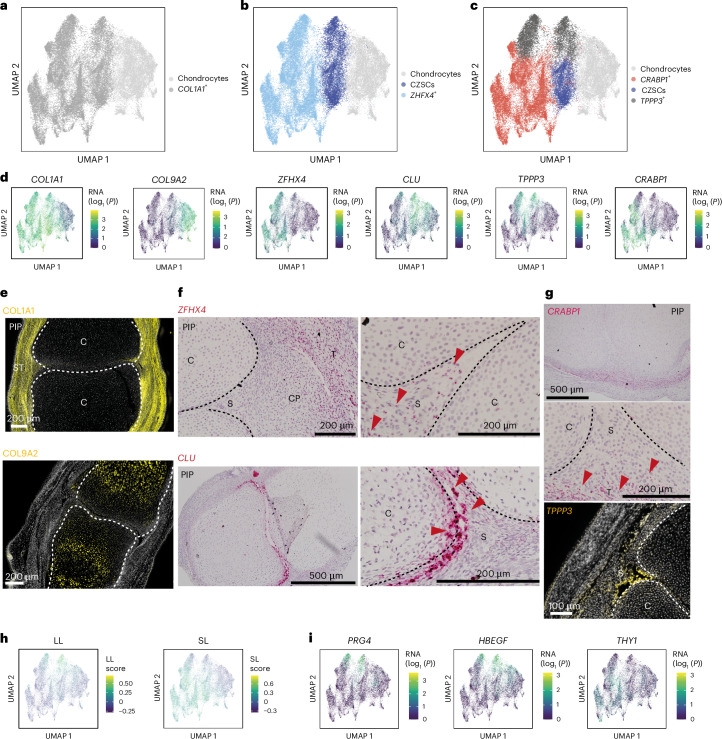
The transcriptional profile of stromal populations in developing joints reflects their spatial location. **a**–**c**, UMAP embeddings displaying stromal cells from all donors and age groups. Clusters were grouped into stromal zones based on expression of key markers (see **d**). **d**, UMAP embedding displaying RNA expression of key markers used to identify stromal zones. **e**–**g**, Immunofluorescence and RNAscope images showing the spatial location of markers defining stromal zones in 14–15 pcw PIP joints. Representative images from three donors are shown; ST, soft tissue; C, cartilage; CP, capsule; S, synovium; T, tendon. **h**, UMAPs of developmental stromal cells, scored by their expression of LL and SL gene signatures. LL and SL signatures were generated using the top 100 markers from a published RA single-cell data set^14^. **i**, UMAP embedding displaying expression of key LL (*PRG4*, *HBEGF*) and SL (*THY1*) markers.

**Extended Data Fig. 2:**
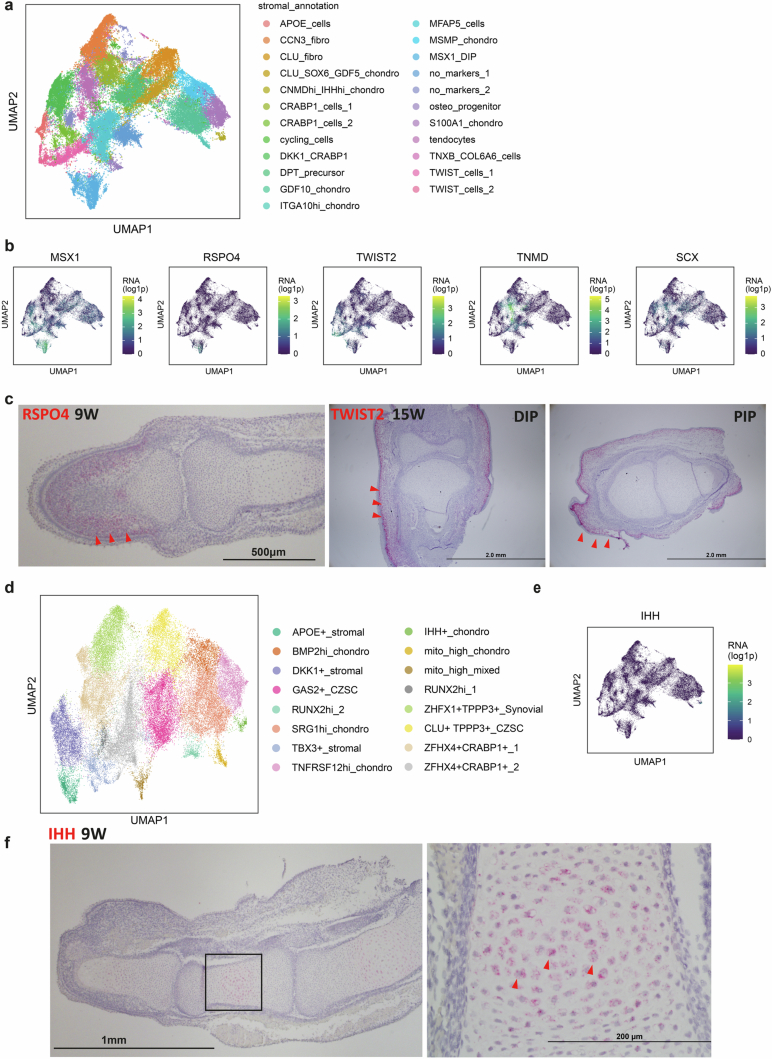
Transcriptional and spatial characterisation of all stromal populations. (**a**) UMAP embedding and cluster assignments based on stromal cells only. (**b**) Feature plots of stromal embedding, displaying expression of markers used to identify juxta-articular contaminating populations. (**c**) RNAscope showing the spatial location of RSPO4+ and TWIST2+ cells in 9pcw and 15pcw developing joints. RSPO4+ cells are primarily found in the finger-tip while TWIST2+ cells were abundant in the skin. Images representative of n = 2 donors. (**d**) UMAP embedding and cluster assignments for stromal cells with peripheral populations removed. 16 clusters were identified. (**e**) Feature plot displaying expression of IHH. (**f**) RNAscope showing the location of IHH+ cells. Red arrows indicate positive cells. Images represent n = 2 donors.

**Extended Data Fig. 3:**
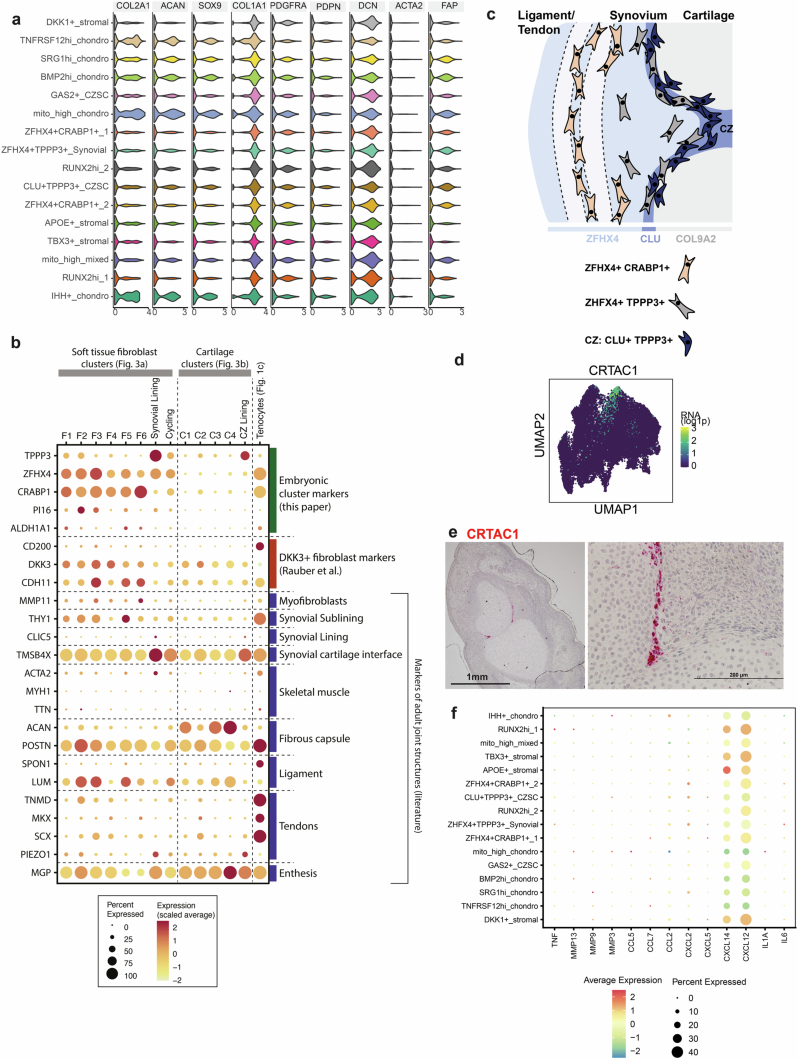
Further characterisation of stromal populations, including expression of known markers for cell types and anatomical structures. (**a**) Violin plots showing expression of chondrocyte and fibroblast markers across stromal populations. (**b**) Dotplot showing average gene expression and percentage of cells with detection in our STF clusters and tenocytes. The markers and their sources are described in Supplementary Table 11. They comprise of embryonic markers from this publication, adult cell type markers as well as literature markers for relevant anatomical structures. (**c**) Schematic of stromal zones in the developing joint. (**d**) Feature plots showing the restricted expression of CRTAC1 in the synovial and CZ lining. (**e**) RNAscope images showing the location of CRTAC1+ cells in 14-15pcw developing joints. Representative images from n = 3 donors are shown. (**f**) DotPlot showing expression of inflammatory and damage associated genes across stromal populations. CZ=cartilage zone.

To investigate the transcriptional identity of stromal cells in developing joints, we reanalyzed stromal cells separately. Based on the results of in situ histology, we removed a small number of contaminating juxta-articular cells (Extended Data Fig. 2a–c). Analysis of the remaining cells identified 16 stromal clusters (Extended Data Fig. 2d) that could be grouped into three distinct regions, reflecting their anatomical location. These comprised (1) *COL1A1*^+^*ZHFX4*^+^ ‘soft tissue fibroblasts’ (STFs; encompassing ligament/capsule and synovial fibroblasts), (2) *COL1A1*^+^*CLU*^+^ ‘cartilage zone stromal cells’ (CZSCs) and (3) *COL9A2*^+^ chondrocytes (Fig. 2a–c). The identified STF and CZSC/chondrocyte clusters did not show obvious expression of known markers of adult extra-articular tissues such as tendons or skeletal muscle (Extended Data Fig. 3b). These findings suggest that spatial cues are important in sculpting transcriptional identity.

Immunofluorescence demonstrated that COL1A1, which marks both *ZHFX4*^+^ STFs and *CLU*^+^ CZSCs, was expressed in the soft tissue surrounding the joint and formed a thin layer across the articular surface (Fig. 2e), whereas COL9A2 was restricted to the cartilaginous anlagen (Fig. 2d,e). This region encompasses distinct chondrocyte subsets, a *RUNX2*^+^ population that expresses osteoblast genes and an *IHH*^+^ subset located in the center of the anlagen (Extended Data Fig. 2e,f). *CLU*^+^ CZSCs were named according to their location adjacent to cartilage, where they bridged synovium and cartilage structures and ran along the articular surface (Fig. 2d,f). They expressed genes associated with both fibroblasts (*COL1A1*, *FAP*, *PDGFRA* and *PDPN*) and chondrocytes (*SOX6* and *SOX9*; Extended Data Fig. 3a) but lacked expression of mature cartilage markers (*COL2A1* and *ACAN*). This population could include a specialized protective layer of articular cartilage, as described in fetal mouse and human joints^10,11^.

*ZFHX4* was broadly expressed by fibroblasts outside *CLU*^+^ CZSCs and *COL9A2*-expressing chondrocyte populations. In situ imaging demonstrated *ZFHX4* expression in the synovium and surrounding joint soft tissue, consistent with it marking fibroblasts of the synovium and capsule/ligaments (Extended Data Fig. 3b). *ZFHX4*^+^ STFs could be further divided into *CRABP1*^+^ and *TPPP3*^+^ fibroblasts (Fig. 2c,d). RNAscope revealed highest expression of *CRABP1* in extrasynovial structures, such as ligaments and capsule, whereas detection in the synovium was less frequent. Conversely, the highest levels of TPPP3 were restricted to the synovium and cartilage cell layers that lined the developing joint cavity (Fig. 2g). This suggests that *ZFHX4*^+^*CRABP1*^+^ STFs include fibroblasts in ligaments/capsule and early SL fibroblasts, whereas *TPPP3*^+^ STFs represent synovial lining fibroblasts (Fig. 2g). TPPP3, a marker of joint cavitation in mice^12^, was also expressed by a subset of CZSCs, in keeping with their similar location adjacent to cartilage. At the developmental stages examined, tendons, ligaments and the synovium have not fully developed into separated structures. Thus, we assigned STFs to spatially distinct joint regions that prefigured these structures (Extended Data Fig. 3c).

### Cells at the synovial–cartilage interface develop an LL phenotype

Adult synovium is composed of two main functional compartments: the LL that is in contact with the articular cavity, which produces lubricants, and the SL, where areolar tissue and neurovascular cells are located^13^. Therefore, we next determined whether distinct LL and SL stromal phenotypes exist in developing joints. Gene signatures for LL and SL fibroblasts were generated from a publicly available scRNAseq dataset^14^ and were used to score our embryonic joint atlas (Fig. 2h). In both the STF and CZSC regions, the LL signature mapped onto *TPPP3*^+^ cells, which expressed the canonical LL markers *PRG4* and *HBEGF* (Fig. 2i).

The marker *CRTAC1* uniquely identified the CZSC lining, distinguishing this subset from the STF lining (Extended Data Fig. 3d). *CRTAC1* was specifically expressed at the interface between the synovium and cartilage (Extended Data Fig. 3e), but, unlike the broader CZSC marker *CLU*, *CRTAC1* was not located across the articular surface. Thus, the LL phenotype may represent a polarized cell state that can be acquired by both classical fibroblasts and chondrocyte-like cells. By contrast, the SL signature was more broadly expressed across STFs (Fig. 2h). Unlike their RA counterparts, these embryonic populations displayed low expression of genes associated with inflammation or joint damage (Extended Data Fig. 3f).

### LL cells are predicted to arise from multiple cell lineages

**Fig. 3:**
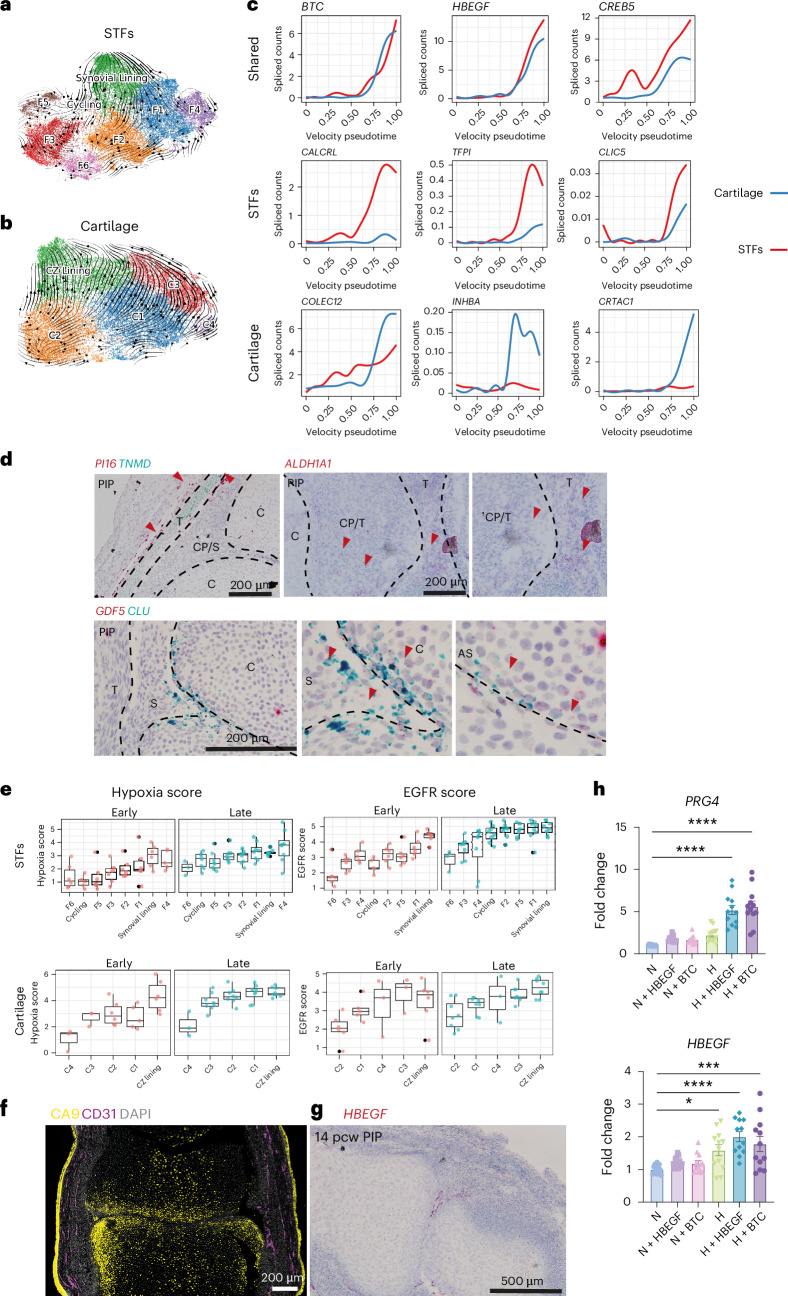
STF and cartilage zone lining populations may develop from different origins, driven by a combination of hypoxia and EGF ligands. **a**,**b**, UMAP embeddings displaying STF (**a**) and cartilage cells (**b**), including SFT and cartilage-specific clustering and RNA velocity vectors. **c**, Generalized additive models fitting gene velocity trends along pseudotime extracted from fibroblast (F1/F2/F4 lining) and chondrocyte (C2–CZ lining) trajectories toward the LL. **d**, RNAscope showing the location of synovial and cartilage progenitors, based on expression of *PI16*, *GDF5* and *ALDH1A1* (red and blue dots). Images represent *n* = 3 donors at later stages (12–14 pcw); AS, articular surface. **e**, Box plots showing Progeny scores for hypoxia and EGFR signaling across fibroblast and cartilage populations. Scores were generated with pseudobulked data for each donor; early = 8–9 pcw (*n* = 6 joints from *n* = 3 donors), late = 12–14 pcw (*n* = 8 joints from *n* = 4 donors). The center lines represent the median, boxes show the interquartile range, and whiskers extend to the highest and lowest values within 1.5× the interquartile range; dots represent joints. Data points outside the whiskers are plotted individually as outliers. Colored dots represent a donor. **f**,**g**, Immunofluorescence (**f**) and RNAscope images (**g**) showing expression of the hypoxia marker CA9 (yellow) and *HBEGF* (red dots). **h**, Gene expression of *PRG4* and *HBEGF*, measured by real-time quantitative PCR (qPCR), in fibroblasts isolated from developing PIP and DIP joints. Cells were cultured under normoxic (N) or hypoxic (H) conditions (1% O_2_, H) ± BTC or HBEGF. Fold change in expression is displayed ${2}^{-\varDelta \varDelta {C}_{{\rm{t}}}}$; change in cycling threshold (target–house keeping gene) for treated samples, relative to control samples; *n* = 6 donors. Each dot represents two replicates per donor, and error bars represent s.e.m. Statistical significance was tested using a one-way analysis of variance with a Dunnett’s multiple comparison test. For *PRG4* N versus H + HBEGF *P* ≤ 0.0001 and for N versus H + BTC *P* ≤ 0.0001. For HBEGF N versus H *P* = 0.017, for N versus H + HBEGF *P* ≤ 0.0001 and for N versus H + BTC *P* = 0.0009; *P < 0.05; ***P < 0.001; ****P < 0.0001.

**Extended Data Fig. 4:**
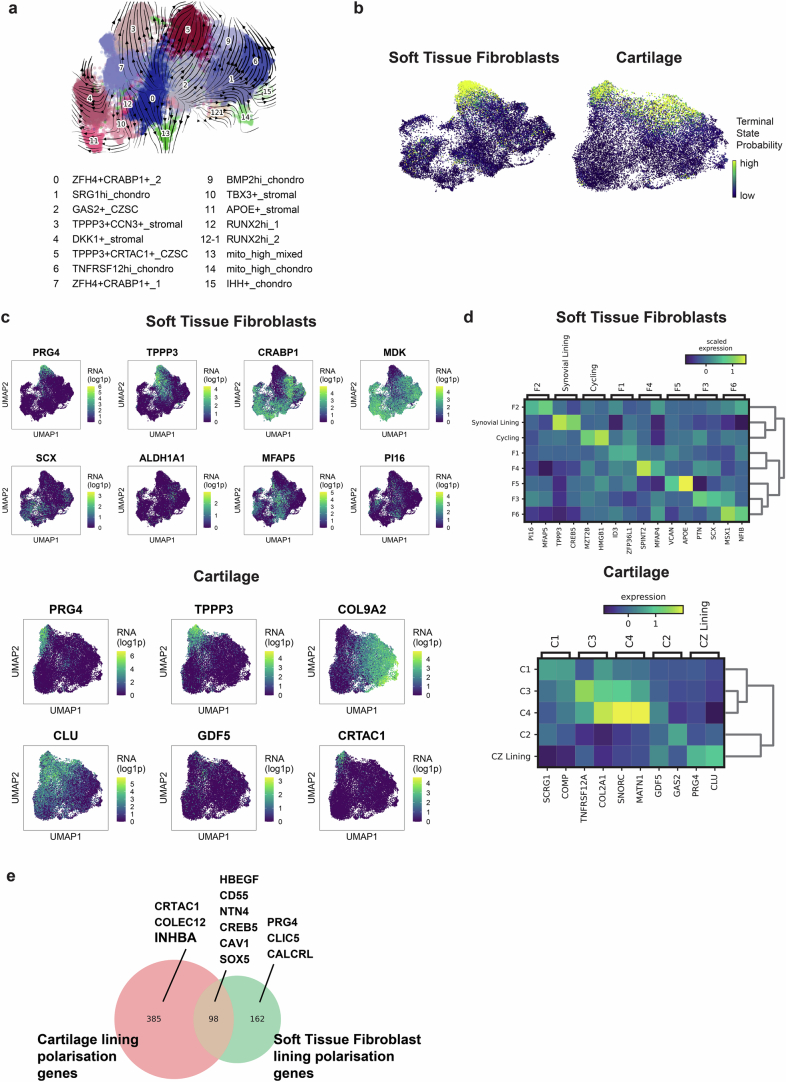
RNA velocity on all stromal populations, marker expression across STF and cartilage populations and shared and unique markers in LL populations. (**a**) UMAP showing stromal embedding and clusters with RNA velocity vectors. (**b**) Absorption probabilities from CellRank for terminal end states in Fibroblast and Cartilage embeddings. (**c**) UMAP embeddings showing expression of key markers defining new STF and cartilage clusters. (**d**) Scaled expression of selected markers across STF (top) and cartilage (bottom) populations. (**e**) Venn Diagram displaying number of overlapping and unique lining trajectory-fitting genes, between the CZ and synovial lining. The overlap is statistically significant (OR 55.29, p = 4.25 × 10^−103^, Two-sided Fisher’s exact test) compared to random chance.

In keeping with the concept that LL cells may arise from different developmental lineages, diverging RNA velocity^15^ streams were identified within STF and CZSC/chondrocyte populations (Extended Data Fig. 4a). Therefore, to dissect their cellular origins and differentiation trajectories in more detail, we reanalyzed STF- and cartilage-associated cells (CZSCs and chondrocytes) separately (Fig. 3a,b).

**Extended Data Fig. 5:**
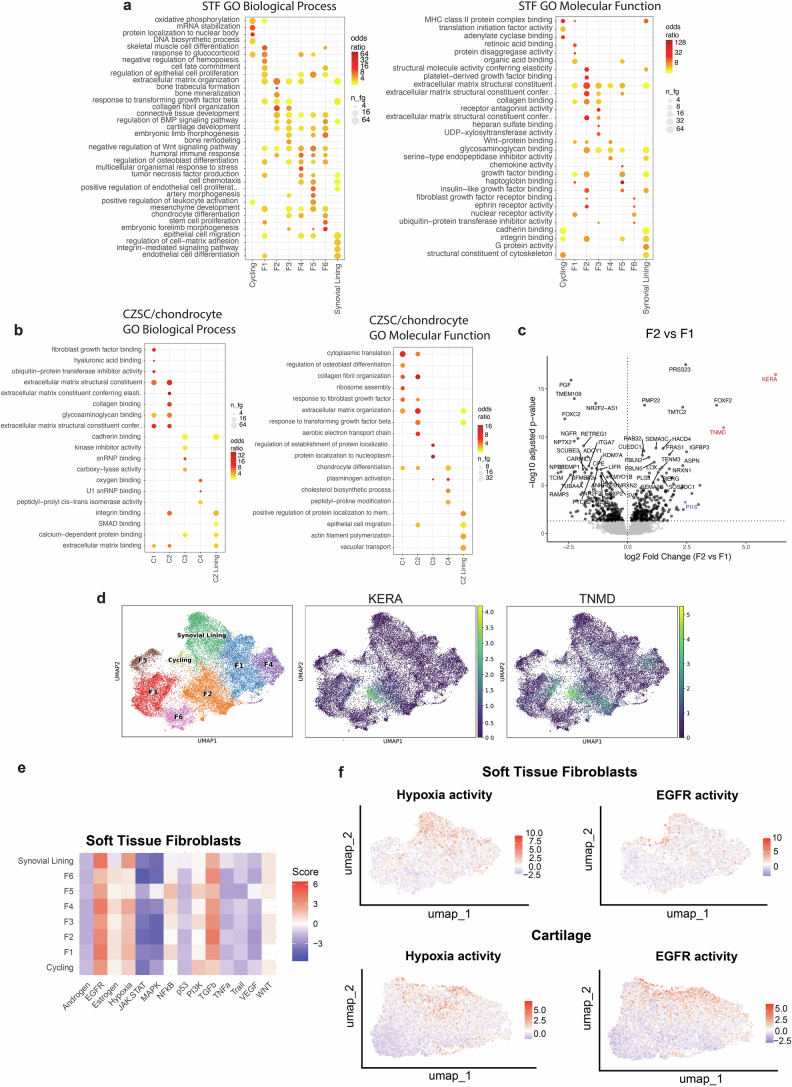
Detailed transcriptomic characterisation of STF and cartilage clusters. (**a**,**b**) Over-representation analysis was performed on the top markers from STF clusters and CZSC/chondrocyte clusters. Selected GO pathways for biological process (**a**, left panel) and (**b**, left panel) and molecular function pathway (**a**, right panel) and (**b**, right panel) are shown. Gene set over-representation significance was identified using the *gsfisher* package and defined as BH-adjusted p-value < 0.05. n_fg = number of foreground genes. (**c**) Volcano plot showing pseudobulk differential expression between F2 (PI16^+^) and F1 (ALDH1A1^+^) clusters (DESeq2 pseudobulk analysis; n = 7; Wald test (two sided); significant defined as BH-adjusted p < 0.05). Significant genes are represented by black dots; top 50 by adjusted p-value labelled, including tendon-associated genes in red. (**d**) Expression of KERA and TNMD in the STF embedding, highlighting their presence in a subset of F2 cells. (**e**) Heatmap displaying predicted activity of different signaling pathways, calculated using the R package progeny, in STFs. (**f**) UMAP embeddings displaying progeny scores for Hypoxia and EGFR signaling activity, at the single cell level, in STF and cartilage embeddings.

Clustering of STFs alone identified eight subpopulations, including a *TPPP3*^+^*PRG4*^+^ lining cluster and seven *CRABP1*^+^ clusters (F1–F6). Within *CRABP1*^+^ fibroblasts, F3, F5 and F6 subsets expressed higher levels of *CDH11* (Extended Data Figs. 3b and 4c,d), whereas F1 and F2 were identified by expression of *ALDH1A1* and *PI16*, respectively (Extended Data Fig. 4d), although *ALDH1A1* was also weekly expressed in clusters F5 and F6 (Extended Data Fig. 3b). Gene set over-representation analysis revealed an enrichment for the ‘response to transforming growth factor-β (TGFβ)’ pathway in the F1, F2 and synovial lining clusters, consistent with its known role in synovitis and synovial lining proliferation (Extended Data Fig. 5a and Supplementary Table 12)^16^. The F2 marker *PI16* is characteristic of the ‘universal fibroblast’, a multipotent fibroblast progenitor that is present in multiple tissues^17^. In situ RNA expression revealed that *PI16*^+^ fibroblasts resided at the border of tendons and ligaments, as well as around possible vascular structures (Fig. 3d). Consistent with this location, pseudobulk differential expression analysis of the F1 and F2 clusters identified a subregion of the *PI16*^+^ F2 cluster that expressed the tendon markers *TNMD* and *KERA* (Extended Data Fig. 5c,d and Supplementary Table 13).

Analysis of CZSCs/chondrocytes identified three *COL9A2*^+^ chondrocyte subpopulations (C1, C3 and C4) and two *CLU*^+^ CZSC clusters comprising *TPPP3*^+^*PRG4*^+^ LL cells and the *CLU*^+^*PRG4*^*−*^ C2 subset (Extended Data Fig. 4c,d). Despite loss of interzone structures at these developmental stages, CZSCs also expressed *GDF5* (ref. ^8^), suggesting that these cells may be interzone descendants. In keeping with this concept, in situ RNA analysis showed *GDF5* expression within the *CLU*^+^ articular surface and synovial LL (Fig. 3d). Gene set over-representation analysis showed enrichment for pathways involved in ‘chondrocyte differentiation‘, ‘response to fibroblast growth factor’ and ‘response to TGFβ’ (C1 and C2), consistent with a chondrocyte fate, along with an enrichment for ‘cadherin binding’ (C3), which is an essential event in chondrocyte maturation (Extended Data Fig. 5b and Supplementary Table 13)^18^.

CellRank analysis^19^ predicted that both the STF and CZSC lining populations represent terminal states (Extended Data Fig. 4b). Velocity vectors indicated that the CZSC lining originated from the *PRG4*^−^ portion of the CZSC, which also gives rise to *COL9A2*^+^ differentiated chondrocytes, whereas the STF lining was predicted to originate from the *PI16*^+^ F2 and *ALDH1A1*^+^ F1 STF populations. In addition to synovial lining, velocity vectors also suggested that F2 *PI16*^+^ cells differentiate into *ALDH1A1*^+^ F1 and *CDH11*^+^ F6 fibroblasts, in keeping with their multipotent potential in adult tissue^17^. Together, these data suggest that the LL is derived from multiple developmental origins, representing a polarized state that can be acquired by different cellular lineages.

### Spatial cues driven by EGF signaling and hypoxia mediate LL polarization

To identify genes associated with differentiation toward a lining state, we analyzed the two major predicted differentiation trajectories (from F1 and F2 to lining for the STF lineage and from C2 to the CZSC lining for the cartilage lineage). Ninety-eight genes were common to both trajectories, indicating the presence of a common differentiation program (odds ratio = 55.29, *P* = 4.25 × 10^−103^ compared to random chance; Extended Data Fig. 4e). These genes included the canonical LL marker *HBEGF* and the transcription factor *SOX5*. Never-the-less, we also noted the existence of genes specific to the STF lineage (*n* = 162 genes, including *CALCRL*) or CZSC lineage (*n* = 385 genes, including *CRTAC1* and *INHBA*; Fig. 3c and Extended Data Fig. 4e).

The predicted polarization of multiple cell lineages toward a common *SOX5*^+^*HBEGF*^+^ LL phenotype suggests regulation by a shared morphogenic cue at the synovial–cartilage interface. Progeny^20^ analysis identified higher activity of the hypoxia, EGFR and TGFβ signaling pathways within embryonic STFs (Extended Data Fig. 5e). The activity of the hypoxia and EGFR pathways was higher in both STF and CZSC lining populations than in other clusters (Fig. 3e and Extended Data Fig. 5e,f). Expression of *HBEGF* and *BTC* by LL cells in combination with the location of *HBEGF* transcripts and the HIF-1 target CA9 at the synovial–cartilage interface suggested that these are potential candidates for LL induction (Fig. 3f,g). To test this, fibroblasts were isolated from fetal finger joints and cultured under hypoxic conditions with or without HBEGF and BTC. Strikingly, combined hypoxia and EGF ligand stimulation significantly increased expression of the LL markers *PRG4* and *HBEGF* (*P* = 0.009 and *P* = <0.0001, analysis of variance with a Dunnett’s multiple comparisons test; Fig. 3h).

**Extended Data Fig. 6:**
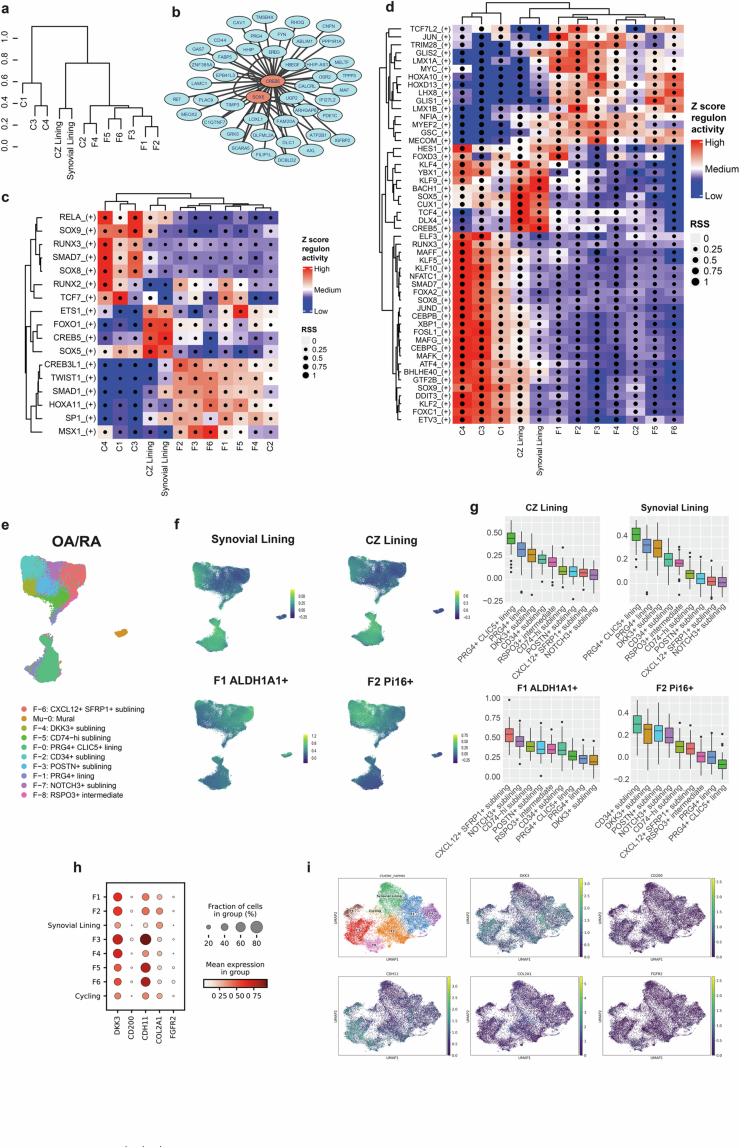
STF and Cartilage regulon activity and further comparisons of adult and embryo stromal populations in the joint. (**a**) Hierarchical clustering of STF and cartilage clusters based on transcriptional activity as indicated by 344 regulons detected in the joint dataset. (**b**) Predicted gene regulatory network associated with STF lining, constructed using pySCENIC and Cytoscape, showing transcription factors SOX5 and CREB5 and significantly associated target genes. (**c**) Heat map/dot-plot showing row-scaled activity of selected enriched regulons (colour scale) and respective cell-type specificity (RSS) as dot sizes. (**d**) Heat map/dot-plot showing row-scaled activity of the top 50 most active regulons across the dataset on a color scale and cell-type specificity (RSS) of the regulon as dot sizes. (**e**) UMAP embedding and fibroblast populations from NIH AMP2 data (RA patients = 73, OA patients = 9). (**f**) UMAP showing module scores of gene signatures (top 100 markers) from embryonic clusters. (**g**) Box plots display embryonic gene scores in adult data pseudobulked by donor and cluster. Centre line represents the median, box shows the interquartile range, whiskers extend to highest and lowest values within 1.5x the interquartile range and dots represent sample outliers outside 1.5x the interquartile range. Dot plot (**h**) and UMAPs (**i**) displaying expression of genes associated with CD200+ fibroblasts in embryonic single cell data.

We next examined gene regulatory networks using pySCENIC^21^ and generated a map of transcription factor activity and the corresponding regulated gene modules (regulons) across clusters. This confirmed that the LL clusters shared regulatory networks (Extended Data Fig. 6a), including *SOX5*, *CREB5*, *ETS1* and *FOXO1*, consistent with data from the mouse lining^22^. Furthermore, the *SOX5* and *CREB5* regulatory networks (Extended Data Fig. 6b–d) highlighted interactions with lining genes (*PRG4*, *TPPP3* and *CAV1*), including those associated with EGFs (*HBEGF* and *EREG*), supporting the role for EGF ligands in specification of the lining phenotype.

### Spatially and functionally distinct fibroblast populations in adult RA are laid down during embryonic development

We next investigated how stromal populations from developing finger joints relate to pathogenic fibroblast subsets in adult arthritis using the Accelerated Medicines Partnership phase II (AMP2) dataset (Extended Data Fig. 6e)^6^.

**Fig. 4:**
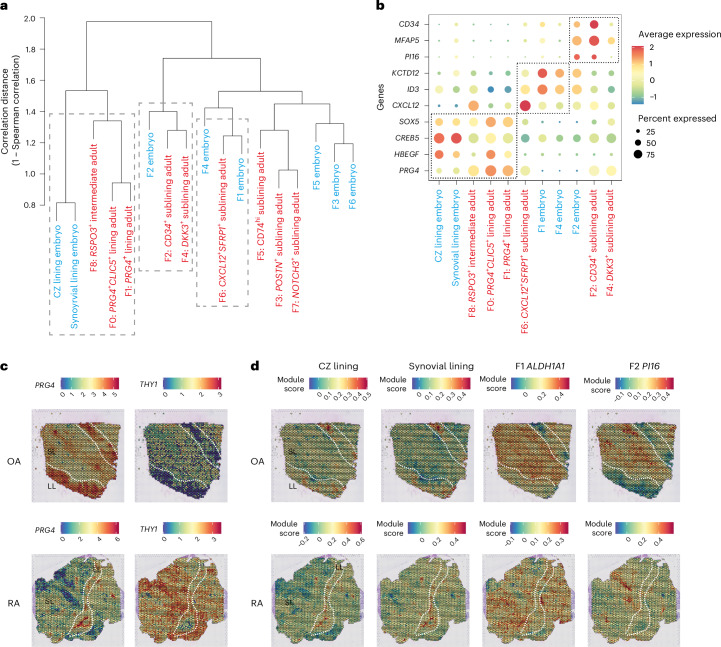
Embryonic stromal populations are homologous to spatially distinct adult fibroblasts in OA and RA. **a**,**b**, Comparison of embryonic and adult OA and RA fibroblasts (NIH AMP2 data; individuals with RA = 73, individuals with OA = 9). **a**, Hierarchical clustering based on Spearman correlation of the top 100 markers, showing transcriptionally homologous populations. **b**, Dot plot displaying expression of gene cassettes shared between healthy embryonic and adult OA/RA cell populations. **c**,**d**, Visium spatial transcriptomics data of OA and RA synovium (*n* = 6 OA donors, *n* = 11 established RA donors, *n* = 6 early RA donors, *n* = 5 resolving RA donors). **c**, Representative images of *PRG4* (LL) and *THY1* (SL) expression in OA and RA synovium. **d**, Representative images of OA and RA synovium, scored according to their expression of gene signatures (top 100 markers) from embryonic fibroblast and cartilage zone clusters. White dotted lines indicate separation between LL and SL.

Analysis of marker gene similarity showed that embryonic STF and CZSC lining subsets clustered with the adult lining in arthritis, sharing expression of key marker cassettes (*PRG4*, *HBEGF*, *CREB5* and *SOX5*; Fig. 4a,b). Conversely, the fetal F2 *PI16*^+^ and F1 *ALDH1A1*^+^ populations clustered with adult F2 *CD34*^+^ and F6 *CXCL12*^+^*SFRP1*^+^ SL populations, respectively (Fig. 4a,b). Fetal F2 *PI16*^+^ and adult F2 *CD34*^+^ populations shared expression of *PI16*, *MFAP5* and *CD34*, whereas fetal F1 *ALDH1A1*^+^ and adult F6 *CXCL12*^+^ cells both expressed *KCTD12* and *ID3* (Fig. 4b). The existence of transcriptionally homologous synovial stromal cell populations in fetal (finger) and adult (knee/wrist) joints was confirmed by visualization of the expression of cluster-specific gene modules (Extended Data Fig. 6f,g). We also explored whether embryonic F4 cells related to recently described proresolving cells CD200^+^DKK3^+^ fibroblasts in adult arthritis^23^. *DKK3* expression was diffuse across embryonic fibroblasts, and *CD200* expression was minimal, suggesting limited correspondence (Extended Data Fig. 6h,i).

Spatial mapping of embryonic gene signatures in adult disease was also performed by scoring Visium spatial transcriptomics of RA and OA tissue (RA: *n* = 22, OA: *n* = 6). Both CZSC and STF lining signatures were enriched in the lining compartment of OA and RA tissue, where they occupied similar spatial niches (Fig. 4c,d). By contrast, developmental F1 *ALDH1A1*^+^ and F2 *PI16*^+^ populations map to discrete regions of the synovial SL, a niche associated with pathogenic expansion in RA. Overall, these data suggest that functionally and spatially unique fibroblast populations in disease are specified during embryonic development.

### PI16^+^ synovial progenitors are enriched in PIP compared to DIP joints

**Fig. 5:**
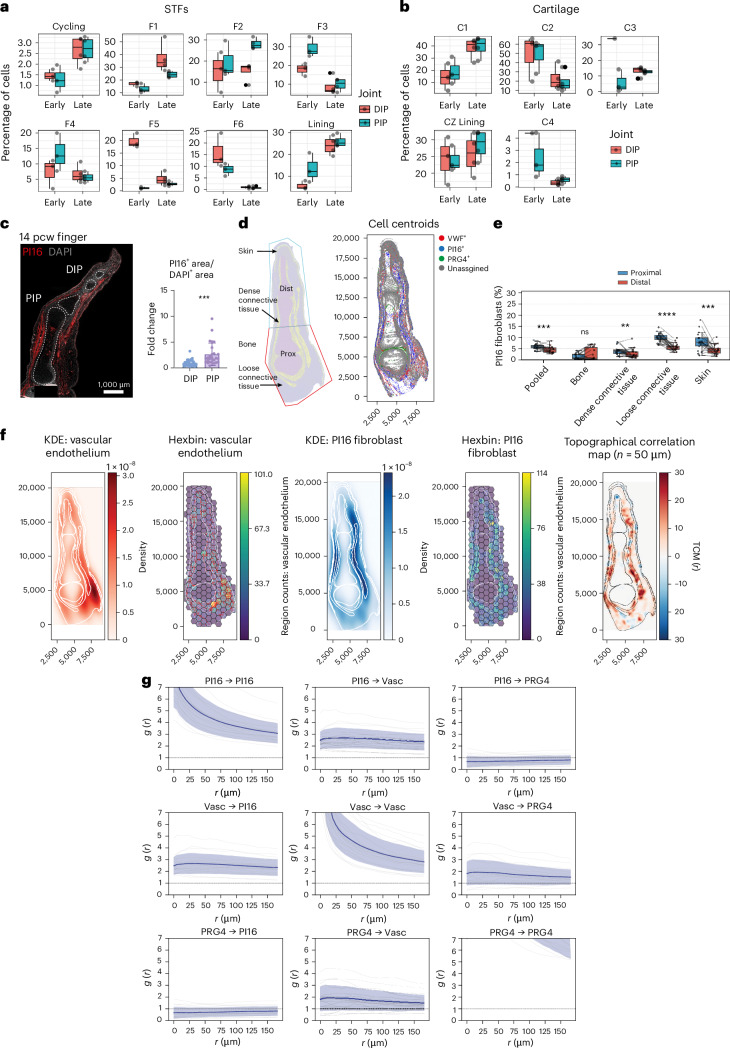
*PI16*^+^ fibroblasts are enriched in PIP joints and occupy distinctive spatial niches. **a**,**b**, Percentages of STF (**a**) and cartilage (**b**) populations within the DIP and PIP joints (percentage of respective embedding; early: 8–9 pcw, *n* = 3 donors; late: 12–14 pcw, *n* = 4 donors). The center lines represent the median, boxes show the interquartile range, and whiskers extend to highest and lowest values within 1.5× the interquartile range. Data points outside the whiskers are plotted individually as outliers. **c**, QuPath quantification of PI16 staining across serial finger sections (14–15 pcw, *n* = 3 donors, 10, 8 and 6 sections per donor). Left, representative image of PI16 expression (the dotted line indicates cartilage anlagen). Right, PI16^+^ area (μm^2^) in the tendon, capsule and synovium was normalized to DAPI^+^ area (μm^2^). The bars represent mean fold change ± s.d. in PI16:DAPI ratio (relative to DIP joints) for paired regions; *P* = 0.0005 (two-tailed paired *t*-test). Dots indicate sections. **d**–**g**, A spatial analysis tool and the statics package MuSpaN were applied to serial finger sections. **d**, Representative images of tissue annotations (left) and point cloud (right) showing cell centroids colored by cell annotation; Dist, distal; Prox, proximal. **e**, Number of PI16^+^ cells across serial finger sections in different tissue regions of proximal and distal areas. The center line represents the median, boxes show the interquartile range, and whiskers extend to highest and lowest values within 1.5× the interquartile range. Data points outside the whiskers are plotted individually as outliers (*n* = 3 donors). Lines indicate proximal and distal pairs (pooled, *P* = 0.00048; dense connective tissue, *P* = 0.0015; loose connective tissue, *P* = 1.36 × 10^−17^; skin, *P* = 0.00031). Data were analyzed by two-tailed Wilcoxon test with a Benjamini–Hochberg adjustment. **f**, Left to right, kernel density estimation (KDE) of VWF expression (hexbin colored by the proportion of VWF^+^ cells within that region), VWF^+^ centroids (red) are overlaid; kernel density estimation of PI16 expression (hexbin colored by the proportion of PI16^+^ cells within that region), PI16^+^ centroids (blue) are overlaid; TCM of PI16^+^ cells in proximity to VWF^+^ cells. Red indicates regions in which cells are highly correlated, whereas blue indicates regions where PI16^+^ cells not correlated with VWF^+^ cells. **g**, Cross-PCF for PI16^+^, VWF^+^ and PRG4^+^ cells across serial finger sections (14–15 pcw). Blue lines represent the average across all sections (±s.d. indicated by surrounding light blue area); Vasc, vascular.

**Extended Data Fig. 7:**
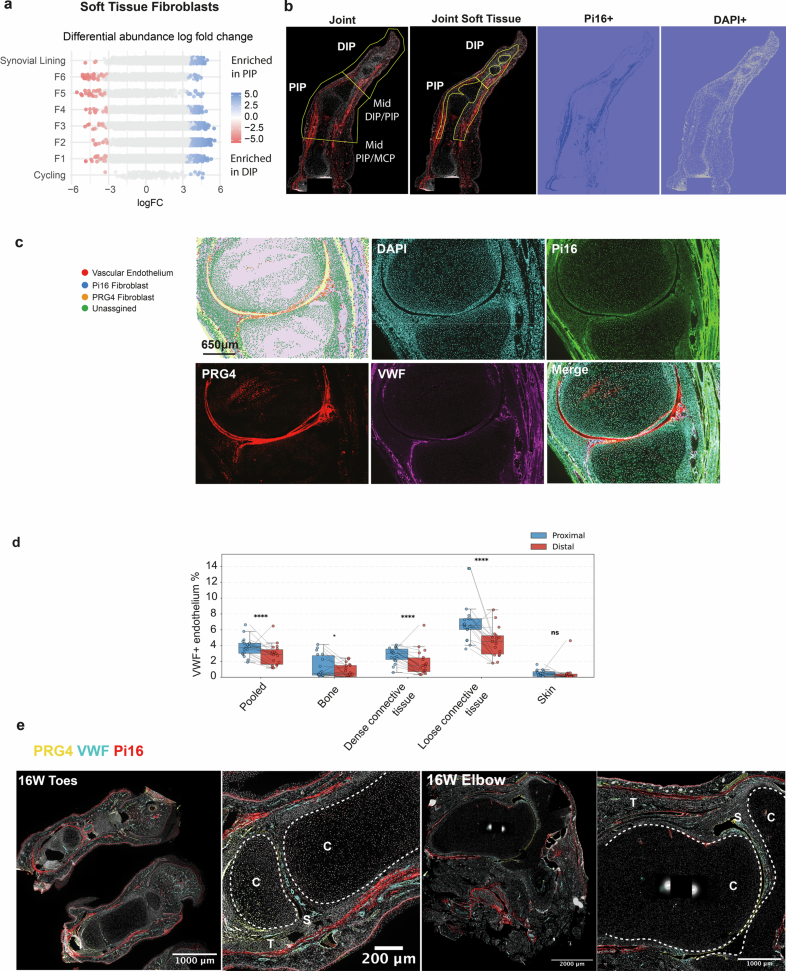
Details on Pi16+ fibroblast niches and spatial data processing. (**a**) LogFC of differential abundance analysis with Milo between joint location (PIP v DIP) for each cell from STF. Cells belonging to abundance neighborhoods at FDR 10% are colored. (**b**) QuPath quantification of Pi16 expression in the DIP and PIP joints. (left-right) Representative images of regions defined as DIP and PIP and ‘joint soft tissue’, regions determined as Pi16+ or DAPI+ using pixel classifiers (Pi16 + =dark blue, DAPI + =grey). Joint soft tissue was manually defined within in the set DIP and PIP regions and includes tendons, ligaments, capsule and synovium. (**c**) Input and output images (depicting a PIP joint) for our bespoke image analysis pipeline. Left to right: Pipeline output; Segmented image with annotated cell types, pipeline input; corresponding raw immunofluorescent staining of DAPI, Pi16 PRG4, and VWF. (**d**) Quantification of VWF+ cells across serial finger sections, using our bespoke image analysis pipeline, comparing different tissues in the distal and proximal regions of the finger. Centre line represents the median, box shows the interquartile range, whiskers extend to highest and lowest values within 1.5x the interquartile range, dots represent a separate image (across n = 3 donors) and lines indicate proximal and distal pairs. Two-sided Wilcoxen test, with a BH adjustment was used to test significance: Pooled; p-adj = 5.63e-5, Dense connective tissue; p-adj=4.63e-5 Loose connective tissue; p-adj=4.17e-7, Bone; p-adj=0.024. *=p-adj < 0.05, **=p-adj < 0.01, ***=p-adj < 0.001, ****=p-adj < 0.0001. (**e**) Immunofluorescent images showing the location of Pi16 fibroblasts in developing elbow and toe joints. n = 2 donors for each anatomical site.

We next investigated whether stromal composition differs between fetal DIP and PIP joints (Fig. 5a,b), which may contribute to their differential susceptibility to certain forms of arthritis. Although the frequencies of some clusters (F5, F3 and C3) varied between joints, their abundance decreased at later time points and may be a product of the proximal–distal nature of finger development. By contrast, at later stages (12–14 pcw), the proportion of *PI16*^+^ F2 progenitors was increased in PIP joints (24.5 ± 7.0% STF cells) compared to in DIP joints (15.2 ± 6.3% STF cells, *P* = 0.00876, paired *t*-test with Bonferroni correction; Fig. 5a). This finding was confirmed by differential abundance testing of the F2 neighborhood using Milo (Extended Data Fig. 7a).

**Extended Data Fig. 8:**
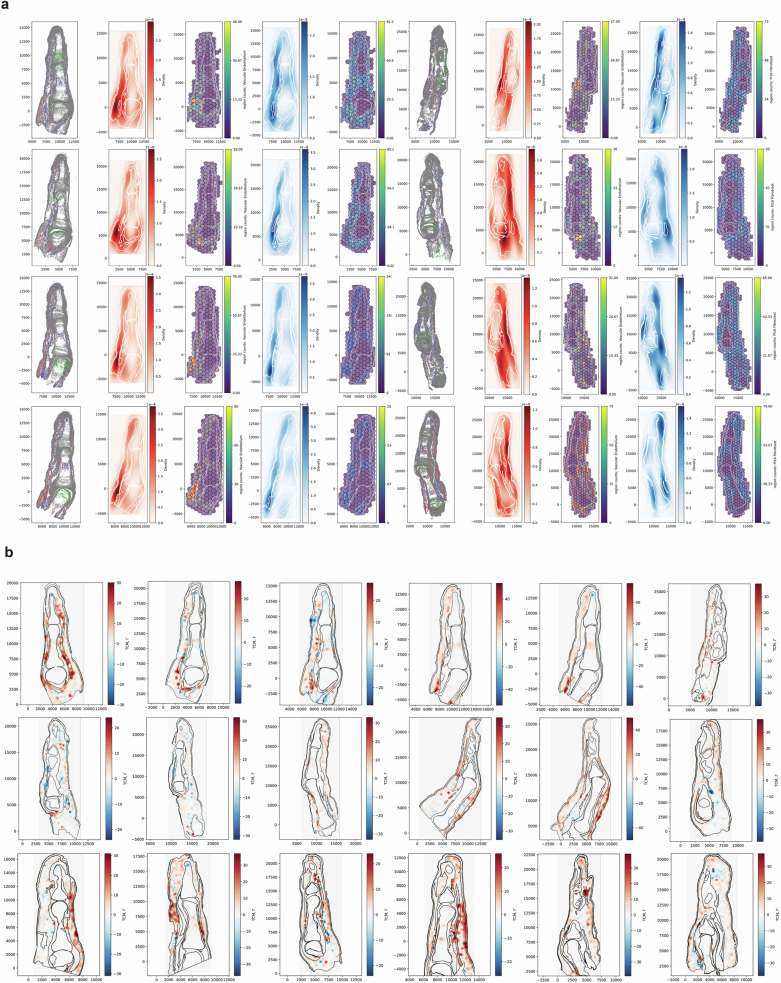
Applying MuSpaN across serial finger sections. Using our image analysis pipeline with the spatial statistics tool MuSpaN. (**a**) Panels showing images across different tissue planes of two fingers from a single donor. Each panel Left to right: Kernel density estimation of VWF expression; hex bin coloured by proportion of VWF+ cells within that region, VWF+ centroids (red) are overlaid; kernel density of Pi16 expression, hex bin coloured by proportion of Pi16+ cells within that region, Pi16+ centroids (blue) are overlaid. (**b**) Panel showing TCM of Pi16+ cells in proximity to VWF+ cells, across different tissue planes of all analysed sections (n = 3 donors). Red indicates regions in which cells are highly correlated, whereas blue indicates regions where Pi16+ cells not correlated with VWF+ cells.

To validate this finding and assess the spatial distribution of these cells, we stained serial sections of developmental fingers for PI16, blood vessel and LL markers VWF and PRG4. The total area of PI16^+^ expression within the joint soft tissue was quantified (Extended Data Fig. 7b), confirming a significant increase in the PIP joint (fold change of 2.63, *P* = 0.0005, paired *t*-test; Fig. 5c). In addition, imaging PI16 across whole finger sections confirmed that *PI16*^+^ fibroblasts are located adjacent to tendon and ligament/capsule borders and in perivascular regions. To characterize the PI16 niche in more detail, we developed a bespoke image analysis pipeline that takes in multiplexed fluorescent images and user annotated histological features to segment^24^, quality control and perform expression-based cell-type annotation. We inputted the aforementioned serial finger sections, along with manual annotations of bone, dense connective tissue (tendons/ligaments), skin and loose connective tissue (tissue that could not obviously be assigned to the previous labels). A spatial map showing the center of each cell in situ (point clouds) was generated, as well as cell annotations based on expression of PRG4, PI16 and VWF (Extended Data Fig. 7c). By incorporating histological labels, we validated an increased percentage of PI16^+^ cells in proximal regions of the finger across different annotated structures (Fig. 5d,e), which appeared to correlate with increased proportions of vasculature in these regions (Extended Data Fig. 7d). Application of a cross-pair correlation function (PCF) from the spatial statics package MuSpAn^25^ demonstrated nonrandom spatial association between PI16^+^ fibroblasts and VWF^+^ blood vessels (*g*(*r*) values > 1), as well as a negative association with PRG4^+^ cells. This supports our earlier findings that *PI16*^+^ fibroblasts are located in vascular regions away from the articular surface (Fig. 5f,g and Extended Data Fig. 8a,b). Topographical correlation maps (TCMs) show the spatial correlation of PI16 and VWF across different planes of the finger. This finding highlighted that PI16^+^ fibroblasts are strongly associated with blood vessels within the interstitial regions bordering the tendons and ligaments (Fig. 5f and Extended Data Fig. 8b) but can also be found independent of the vasculature, suggesting that these cells occupy two spatial niches. This spatial organization was conserved across other joints, including developmental toes and elbows, indicating that *PI16*^+^ cells may play an important role in these niches (Extended Data Fig. 7e). Interestingly, tendons are often affected by inflammation before the onset of RA^26^. Thus, in conjunction with their enrichment in the PIP, *PI16*^+^ fibroblasts appear to be located at sites predisposed to inflammatory arthritis and may help to initiate or propagate inflammation.

### *PI16*^+^ fibroblasts in vitro are linked to tissue organization and show a unique transcriptional response to cytokines

**Fig. 6:**
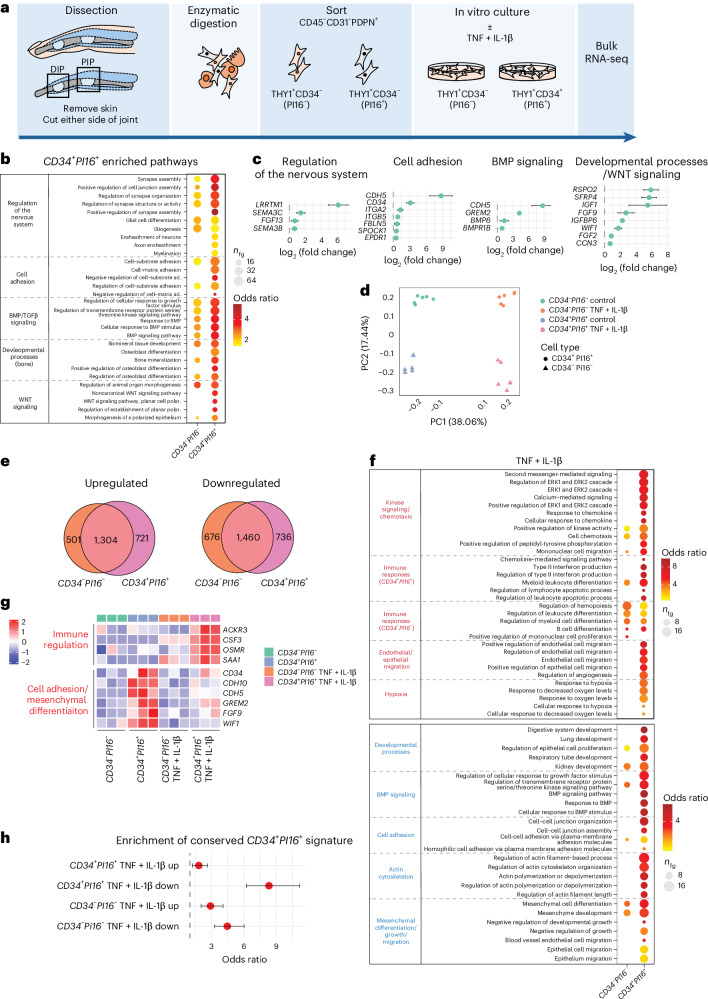
*PI16*^+^ fibroblasts demonstrate a distinct transcriptional response to in vitro stimulation with TNF and IL-1β. **a**, Schematic depicting the experimental design. Fibroblasts were isolated from *n* = 3 independent donors, with *CD34*^+^*PI16*^+^ and *CD34*^−^*PI16*^−^fibroblasts from each donor cultured in duplicate with or without TNF and IL-1β (*n* = 6 samples per condition). **b**, Over-representation analysis was performed on differentially expressed genes between *CD34*^+^*PI16*^+^ fibroblasts and *CD34*^−^*PI16*^−^ fibroblasts, under control conditions. Analogous pathways were clustered based on the similarity of their mapped genes and given an overall functional term (left). Top biological processes for selected clusters are shown (right); ad., adhesion; polar., polarization; *n*_fg_, number of foreground genes. **c**, Plots show the estimated log_2_ (fold change) (DESeq2) in expression of genes mapping to selected Gene Ontology terms. Error bars show 95% confidence interval (log_2_ (fold change) ± 1.96 × s.e.). **d**, PCA of samples using ‘treatment-responsive’ genes only (LRT, adjusted *P* < 0.05). **e**, Venn diagrams show the overlap of differentially expressed genes (treatment versus control) for both *CD34*^+^*PI16*^+^ and *CD34*^−^*PI16*^−^ fibroblasts; left, upregulated genes; right, downregulated genes. Only genes significant in the LRT were included. **f**, Differential gene expression was performed using an interaction term for treatment and fibroblast cell type. Over-representation analysis (gsfisher) with Gene Ontology terms was performed on genes that were up (top) or down (bottom) regulated by TNF + IL-1β and were differentially regulated between *CD34*^+^*PI16*^+^ and *CD34*^−^*PI16*^−^ fibroblasts. Pathways were clustered, and overall ontology terms were assigned (left); most significant pathways (Benjamini–Hochberg-adjusted *P* value) per cluster are displayed. **g**, Heat maps display expression of selected up- and downregulated genes across each donor. **h**, Genes characteristic of PI16^+^ fibroblasts were identified by intersecting genes upregulated by in vitro *CD34*^+^*PI16*^+^ fibroblasts under control conditions and genes upregulated by F2 *PI16*^+^ versus F1–F6 populations from our single-cell data. The plot shows the odds ratio, generated using a two-sided Fisher’s exact test, comparing enrichment of this gene signature in up- or downregulated genes in CD34^+^PI16^+^ fibroblasts and CD34^−^PI16^−^ fibroblasts stimulated with TNF and IL-1β. Error bars show 95% confidence intervals (log_2_ (fold change) ± 1.96 × s.e.). DESeq2 was used to identify differentially expressed genes (two-sided Wald test; significance defined as Benjamini–Hochberg-adjusted *P* < 0.05 and log_2_ (fold change) > 0.5).

**Extended Data Fig. 9:**
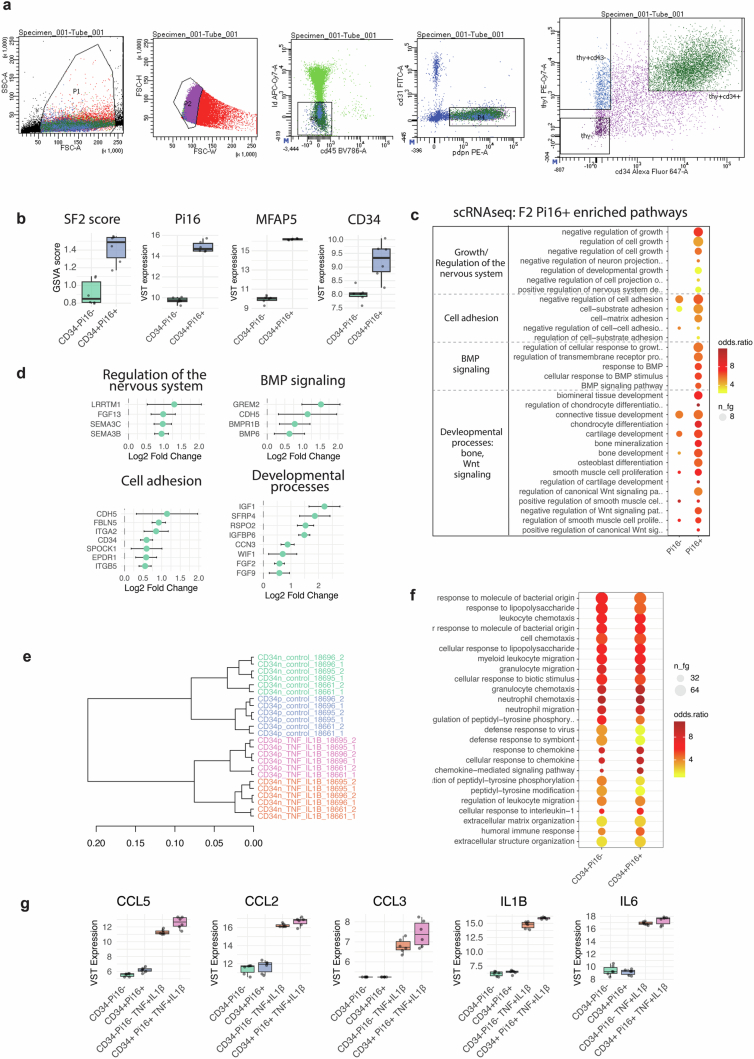
Supporting information for bulk RNA sequencing of cultured *PI16*+ fibroblasts stimulated with TNF and IL1β. Fibroblasts were isolated from n = 3 independent donors, with *CD34* + *Pi16*+ and *CD34-Pi16*- fibroblasts from each donor cultured in duplicate, +/− TNF and IL1β (n = 6 samples/condition). (**a**) Gating strategy for sorting fibroblasts from embryonic joints. Alive, CD45-, CD31-, PDPN + , THY1+ cells were sorted into CD34+ and CD34- populations. (**b**) Boxplots show a GSVA score for top markers from single cell F2 *PI16*+ fibroblasts (left), as well as relative expression (VST) of cassette markers (right) in control *CD34* + *Pi16+* and *CD34-Pi16-* fibroblasts. Centre line represents the median, box shows the interquartile range, whiskers extend to highest and lowest values within 1.5x the interquartile range and dots represent each sample. (**c**) To confirm our *in vitro* finding, DE analysis was performed on pseudobulked F2 *PI16*+ fibroblasts vs *PI16*- fibroblasts (F1-F6) from scRNAseq data. Significant genes were intersected with genes upregulated *in vitro* in control *CD34* + *Pi16*+ fibroblasts. Over-representation analysis (gsfisher) for GO Biological Processes was performed on conserved *PI16*+ genes. Analogous pathways were clustered based on the similarity of their mapped genes and given an overall functional term (left). The top pathways for selected clusters are shown. (**d**) Plots show the estimated log2FC in expression of genes (DEseq2) mapping to ontology terms in *PI16*+ vs *PI16*- fibroblasts from single cell data. Error bars show 95% confidence interval (log2FC ± 1.96 × SE). (**e**) Hierarchal clustering of samples was performed on ‘treatment responsive’ genes (LRT, BH adjusted p value < 0.05). (**f**) DE for both *CD34* + *Pi16*+ *and CD34-Pi16-* fibroblasts (treatment vs control) was performed. Over-representation analysis (gsfisher) with gene ontology terms was performed on upregulated genes and top biological pathways for both populations are shown. (**g**) Boxplots show relative expression (VST) of shared inflammatory factors upregulated in response to TNF + IL1β stimulation. Centre line represents the median, box shows the interquartile range, whiskers extend to highest and lowest values within 1.5x the interquartile range and dots represent each sample. DEseq2 was used to identify DE genes (two-sided Wald test; significance defined as BH adjusted p value < 0.05 and Log2FC > 0.5).

To investigate the response of *PI16*^+^ fibroblasts to inflammatory stimuli and gain insights into their potential role in inflammation, we isolated these cells from embryonic joints for in vitro experiments. Owing to the location of *PI16*^+^ fibroblasts around tendons and extrasynovial structures, skin was removed from fetal fingers, yet tendons and ligaments were left intact (Fig. 6a). PI16 is not a suitable marker for FACS; thus, the coexpressed marker CD34 was used to identify and sort THY1^+^CD34^+^ (SL *PI16*^+^) and THY1^+^CD34^−^ (SL *PI16*^*−*^) populations, hereafter referred to as *CD34*^+^*PI16*^+^ and *CD34*^*−*^*PI16*^*−*^. To test whether cultured *CD34*^+^*PI16*^+^ fibroblasts faithfully recapitulate our single-cell F2 *PI16*^+^ population, a gene set variation analysis (GSVA) score was calculated based on F2 top markers. Expression of this gene signature, in addition to cassette markers *PI16*, *CD34* and *MFAP5*, was higher in *CD34*^+^*PI16*^+^ fibroblasts than in *CD34*^−^*PI16*^−^ fibroblasts (Extended Data Fig. 9b).

Gene set over-representation of differentially expressed genes from control *CD34*^+^*PI16*^+^ fibroblasts (compared to *CD34*^−^*PI16*^−^) demonstrated enrichment of genes encoding proteins involved in structural and spatial organization. These included genes encoding attraction and repulsion cues (*SEMA3B*, *SEMA3C* and *LRRTM1*) and cell adhesion molecules (*CDH5*, *ITGA2* and *ITGB5*; Fig. 6b,c). These cells were also enriched for genes whose products are involved in BMP signaling (*BMP6* and *GREM2*) and bone development, including members of the WNT, FGF and IGF families (*RSPO2*, *SFRP4*, *WIF1*, *FGF9*, *FGF2*, *IGF1* and *IGFBP6*; Fig. 6c). Of note, IGF1, FGF2 and FGF9 have been shown to maintain vascular integrity and support the structural stability of tendons and ligaments^27–29^. We confirmed that these genes and pathways were conserved in vivo by performing the same analysis on differentially expressed genes between single-cell F2 (*PI16*^+^) and F1–F6 (*PI16*^−^) fibroblasts (intersected with genes upregulated by in vitro *CD34*^+^*PI16*^+^ fibroblasts; Extended Data Fig. 9c,d). In combination with their location at developing tendon and ligament boundaries and in the vascular niche, this suggests that *CD34*^+^*PI16*^+^ fibroblasts play a role in establishing and maintaining spatial organization of tissues and in regulating vascular properties.

To test whether *CD34*^+^*PI16*^+^ fibroblasts acquire different properties when exposed to inflammatory cytokines, cells were stimulated with TNF and interleukin-1β (IL-1β). Hierarchical clustering and principal component analysis (PCA) were performed on genes identified as ‘treatment-responsive’ using a likelihood ratio test (LRT) to assess whether cell type influences the effect of cytokine stimulation (Fig. 6d and Extended Data Fig. 8e). Samples primarily separated by treatment (PC1: 38.06% of variance), with additional separation by cell type (PC2: 17.4% of variance). Although most ‘treatment-responsive’ genes were shared between fibroblast populations, a subset of genes were up- or downregulated specifically in different cell types (Fig. 6e). This indicates that the dominant response to treatment is shared, as would be expected on exposure to potent cytokines, yet fibroblast cell type also impacts treatment response. Shared pathways included proinflammatory responses and upregulation of genes encoding inflammatory factors such as *CCL2*, *CCL3*, *CCL5*, *IL6* and *IL1B* (Extended Data Fig. 9f,g).

A differential expression model with an interaction term between stimulation and cell type was used to identify genes whose response to treatment is affected by fibroblast subtype. In addition to shared proinflammatory pathways, fibroblast subsets were also enriched for genes mapping to specific immune programs, including kinase signaling and chemotaxis (*CD34*^+^*PI16*^+^ fibroblasts) and leukocyte and myeloid cell differentiation (*CD34*^−^*PI16*^−^; Fig. 6f,g). Interestingly, pathways downregulated following cytokine stimulation in *CD34*^+^*PI16*^+^ fibroblasts overlapped with those enriched in this cell type under control conditions. This includes BMP signaling and cell adhesion, as well as WNT and FGF family members (*WIF1* and *FGF9*; Fig. 6g). Consistent with these observations, genes upregulated in *PI16*^+^ fibroblasts both in vivo and in vitro, under noninflammatory conditions, were most enriched in genes downregulated by cytokine stimulation in *CD34*^+^*PI16*^+^ fibroblasts (Fig. 6h). This suggests that *CD34*^+^*PI16*^+^ fibroblasts may lose important functional properties in response to inflammatory cytokines. In combination with changes in their immune-regulatory properties, this may disrupt immune signaling, spatial organization and structural integrity in the PI16^+^ niche.

### *PI16*^+^ fibroblasts are found in vascular niches across tissues that develop inflammatory disease

**Extended Data Fig. 10:**
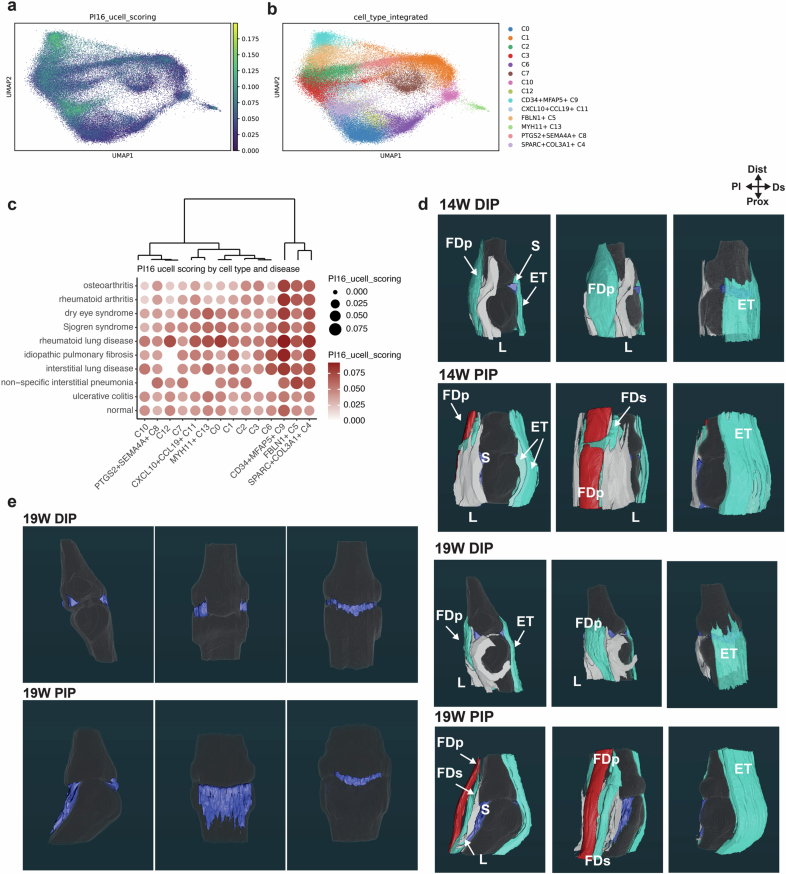
Comparison of Pi16+ fibroblasts from embryonic joints to adult diseases across tissue and soft tissue annotations of DIP and PIP joints using synchrotron x-ray tomography. (**a-b**) Projection of the embryonic PI16 + F2 cluster signature onto an adult cross-tissue fibroblast atlas (Korsunsky et al.), highlighting its distribution across annotated fibroblast clusters. (**c**) Dot plot showing signature expression across fibroblast clusters and conditions in diverse tissues. (**d**) Joint soft tissue structures were manually segmented from tomography scans. 3D projections of 14 and 19pcw DIP and PIP joints are displayed (n = 1 donor). Orientation is indicated by key (top right). Dist = distal, Prox = Proximal, Pl = Palmar, Ds = Dorsal, FDp = Flexor digitorum profundus, FDs = Flexor digitorum superficialis, ET = Extensor tendon, CP = Capsule, S = synovium. (**e**) Synovial (purple) and cartilage structures were manually segmented from tomography scans of 19pcw DIP and PIP joints. Representative 3D projections are shown (n = 1 donor).

As *PI16*^+^ fibroblasts have been identified in different tissues and pathologies, we examined expression of its transcriptional signature in other immune mediated inflammatory diseases using a human cross-tissue fibroblast atlas^30^. The *PI16*^*+*^ fibroblast signature was most enriched in clusters C4 (*SPARC*^+^*COL3A1*^+^, proposed as a universal perivascular fibroblast facilitating immune cell recruitment) and C5/C9 (associated with homeostatic functions and progenitor-like states). Within these clusters, enrichment was highest under inflammatory and fibrotic conditions compared to healthy or degenerative tissues (Extended Data Fig. 10a–c). This suggests that *PI16*^+^ fibroblasts are present in other inflammatory conditions where they may be involved in regulating vasculature niches.

### X-ray tomography confirms differences in soft tissue structures surrounding developing DIP and PIP joints

**Fig. 7:**
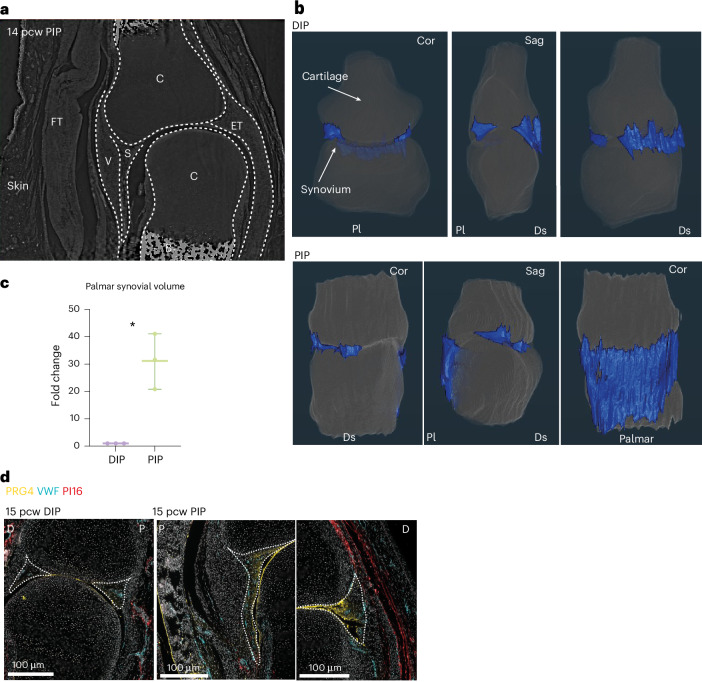
Synchrotron X-ray tomography of developing joints reveals structural differences between DIP and PIP joints. Synchrotron X-ray tomography of 14–15 pcw joints. **a**, Sagittal cross-section of an X-ray image of a 14 pcw PIP joint. Images have been filtered to remove noise and enhance edge contrast. Dotted lines indicate different anatomical structures; FT, flexor tendon; ET, extensor tendon; V, volar plate. **b**, Representative 3D projections are of synovial (blue) and cartilage (gray) structures, manually segmented from tomography scans; Cor, coronal view; Sag, sagittal view; Pl, palmer side; Ds, dorsal side. **c**, Quantification of palmar synovial volume in DIP and PIP joints. Data points show fold change in voxels (±minimum and maximum range) relative to the DIP joint. The center line indicates the mean fold change for each condition; *n* = 3 fingers from *n* = 2 14–15 pcw donors; *P* = 0.036, using a one sample *t*-test (two-tailed). Each dot represents a finger scan; *P < 0.05. **d**, Immunofluorescence images of DIP and PIP joints showing expression of PRG4, VWF and PI16. Images confirming the presence of synovial structures (white dotted line) identified in tomography images are shown. Images represent *n* = 3 14–15 pcw donors; D, dorsal; P, palmar.

Having characterized the cellular composition of DIP and PIP fetal joints, we next investigated their three-dimensional (3D) structure using synchrotron X-ray tomography (Fig. 7a). Segmentation and 3D reconstruction of 14 and 19 pcw joints highlighted known anatomical differences between the PIP and DIP joints (Extended Data Fig. 10d). Although both joints include prominent ligament material (volar plate) on the palmar surface, the PIP joint is surrounded by extra tendon material, arranged in a complex 3D configuration. Two tendons run along the palmar surface: the flexor digitorum profundis (FDP) and the flexor digitorum superficialis (FDS). Proximal to the PIP joint, the FDS splits and attaches to the PIP joint, creating an opening through which the FDP traverses before inserting into the distal phalange. In addition, a striking difference in the synovial structure joints was also observed. In 14–15 pcw DIP joints, the synovium formed a thin layer that directly inserts into the joint space and is more prominent dorsally. Conversely, the synovium of PIP joints formed a large, baggy, apron on the palmar side (Fig. 7b,c), confirmed by 2D imaging (Fig. 7d). Differences in synovial shape were also present at 19 pcw (Extended Data Fig. 10e), suggesting that this is not caused by the developmental delay of the DIP joint relative to the PIP joint and likely reflects the functional specialization of these joints.

## Discussion

By uncovering the cellular composition of developing human finger joints, we demonstrate that the stromal landscape of adult arthritis is sculpted during embryonic development. Spatial distribution and compositional differences in fibroblast subsets, laid down in utero, may explain the differential susceptibility of synovial joints to inflammation.

In fetal joints, fibroblasts and chondrocytes form spatial niches, including *COL9A2*^+^ chondrocytes (anlagen), *ZHFX4*^+^ STFs (tendon, ligament/capsule and synovium) and *CLU*^+^ CZSCs (synovial–cartilage interface). In addition, two *TPPP3*^+^ clusters, both exhibiting an LL phenotype, were predicted to originate from the STF and CZSC lineages. We show that common spatial cues, such as hypoxia and EGF ligands (HBEGF and BTC) may drive polarization toward a shared lining phenotype. Gene regulatory network analysis highlighted that transcription factors *SOX5*, *CREB5* and *ETS1* are central to this process, consistent with a mouse model of cartilage injury^22^.

The CZSC lining appeared to derive from *GDF5*^+^ interzone-like cells, a population unique to the joint. By contrast, the STF lining was predicted to originate from F1 *ALDH1A1*^+^ and F2 *PI16*^+^ fibroblasts, of which the latter resembles the ‘universal fibroblast’, a pan-tissue progenitor^17^. This is supported by murine models, which also suggest that the synovium is derived from interzone and noninterzone origins^22^. Whether different lineages map to functionally or spatially discrete populations in adult arthritis is yet to be determined. However, in murine models of cartilage injury, GDF5-derived LL cells expand and form osteophytes, which contain newly generated cartilage^31,32^. Thus, in response to damage, CZSCs may reactivate progenitor properties, which could be harnessed for cartilage repair. Beyond these inferences, the precise contribution of embryonic stromal subsets to adult joint structures would require lineage tracing, which is not possible in humans.

Our data highlighted an enrichment of *PI16*^+^ fibroblasts in the developing PIP joint. These cells localized to two spatial niches: perivascular regions and the emerging interface of tendons and ligaments, a feature conserved across different joint sites. *PI16*^+^ fibroblasts may act as important adventitial cells having also been observed in the vasculature niche of skin^33^, tonsil^34^, gut and lung^17^. These cells are also found in interstitial tissue of the skin^33^ and breast^35,36^, in skin fascia^37^ and encapsulate the dorsal root ganglion^38^, suggesting they also regulate tissue interfaces.

Tendon inflammation is a common feature of inflammatory arthritis. In fingers, inflammation of flexor, extensor and interosseous tendons occurs early in RA and can predict disease outcome^39–44^. Indeed, flexor tenosynovitis was a stronger predictive factor for persistent disease than the presence of antibodies to citrullinated proteins and articular synovitis^40,42^. This indicates that inflammation of tendons is an early event, which may propagate widespread inflammatory signaling. The distinctive location of *PI16*^+^ fibroblasts at developing tendon borders suggests that they may play a role in tendon inflammation, if also present at this location in adult joints. *PI16*^+^ fibroblasts, both in vivo and in vitro under control conditions, upregulate the expression of genes encoding adhesion molecules, repulsion and attraction cues and factors that regulate vascular and tendon integrity and structure (*IGF1*, *FGF2* and *FGF9*)^27–29,45^. Thus, *PI16*^+^ fibroblasts in homeostasis may play a role in regulating spatial organization and structural integrity at these sites. After exposure to TNF and IL-1β, *PI16*^+^ fibroblasts upregulate a proinflammatory response that is shared with *PI16*^−^ fibroblasts, suggesting that they retain the ability to release proinflammatory factors^1,46^. *PI16*^+^ fibroblasts also show a distinct transcriptional response to cytokine stimulation, including upregulation of genes mapping to chemotaxis and migration pathways. In addition, they also downregulate the expression of genes mapping to their proposed homeostatic functions, such as those encoding adhesion molecules and FGF signaling factors. Thus, under inflammatory conditions, these cells may lose their core properties, consistent with their known role as fibroblast progenitors that differentiate into specialized subsets in disease^17,47^. We hypothesize that these transcriptional changes dysregulate immune responses and disrupt tissue organization and integrity, which may confer increased vulnerability or perpetuate inflammation in the PI16 niche. This is consistent with their reported role in modulating immune responses in tonsils and compromising vascular integrity in the dorsal root ganglion, which enables immune infiltration^34,38^.

Enrichment of *PI16*^+^ fibroblasts in the PIP joint may reflect their complex arrangement of tendon and ligament material, which facilitates increased flexion at this site and enables 85% of the motion required to grasp objects^48–50^. X-ray imaging highlighted known differences in tendon structures along the proximal–distal axis of the finger. On the palmar surface, the FDP alone inserts at the DIP joint, while the PIP joint is surrounded by two intersecting tendons. Although not identified by X-ray imaging, extensor tendons are also known to form an intricate structure around the PIP joint, encompassing a central slip as well as two lateral bands that splinter from the main tendon body. X-ray imaging also revealed differences in synovial structure, highlighting a large baggy pouch on the palmar surface unique to the PIP joint. These synovial structures will experience different mechanical dynamics during finger movement, which may also influence susceptibility to damage and inflammation.

Overall, our findings suggest that joint-specific cellular composition and structural organization established in utero are key determinants of inflammation site specificity, with *PI16*^+^ fibroblasts playing a central role. The presence of *PI16*^+^ fibroblast transcriptional signatures across immune mediated inflammatory diseases suggests that abundance of this population, in combination with native tissue structures, may provide a cellular explanation for inflammation tissue tropism.

## Methods

### Joint dissection and processing

Human embryonic and fetal material was provided by the Joint MRC/Wellcome Trust (MR/X008304/1 and 226202/Z/22/Z) Human Developmental Biology Resource (www.hdbr.org). Joints were processed according to ‘Embryonic joint processing’ in the Supplementary Methods (Supplementary Information).

### 10x Chromium gene expression library preparation and sequencing

Where possible, cells were sorted for viability using 7-AAD dye (Biolegend, 420403) and loaded onto the 10x Genomics Chromium Controller (Chip G) following the manufacturer’s protocol to capture 10,000 cells per reaction. Gene expression libraries were prepared using 10x Genomics Single Cell 3′ Reagent kits (v3.1) following the manufacturer’s user guide (CG000330 Rev B). Libraries were pooled and sequenced on an Illumina NovaSeq6000 system with a customized paired-end, dual-indexing (28,898-base pair) format according to the recommendation by 10x Genomics.

### Alignment and preprocessing of scRNAseq data

The scRNAseq data were aligned and quantified using the 10x Genomics Cell Ranger pipeline (version 4.0.0, human cellranger reference GRCh38 and Ensembl 98, script ‘pipeline_cellranger.py’; https://github.com/sansomlab/tenx/). Quality filtering and initial data processing was performed using the script ‘pipeline_scxl.py’. Cells with more than 10% mitochondrial reads or fewer than 500 genes were excluded. The difference between G2M and S phase was estimated using the expression of known cell cycle marker genes (CellCycleScoring, Seurat v4.3). Doublets were removed using the mean (scrublet score) + 3 s.d. (filter value: 0.26, scrublet v0.2.3)^51^.

For integration, batch correction and dimensionality reduction, we used scVI with correction for mitochondrial content and cell cycle phase (3,000 highly variable genes, 30 scVI latent variables, scVI v0.12.0). The neighbor graph was created from the scVI components using hnsw (implemented by scVelo v0.2.5).

To analyze cell types in more detail, various stages of removing populations, re-embedding and reclustering were performed (detailed in ‘Integration, clustering and annotation of scRNAseq data’; Supplementary Information). The settings used at each stage of subsetting are listed in Supplementary Table 2. Most plots were created in R (v4.1.10) using the packages Seurat (v4.3), SingleCellExperiment (v1.14.1), tenxutils (v0.1; https://github.com/sansomlab/tenx/) and ggplot2 (v3.4.2).

### RNA velocity

Velocyto and scVelo (v0.2.5) were used for RNA velocity analysis. In scVelo, preprocessing included filtering genes with a minimum count threshold of 20 and normalization using scv.pp.filter_and_normalize, followed by scv.pp.moments with default parameters. Gene-specific velocities were estimated with scv.tl.velocity in stochastic mode, and velocity streams were projected onto the graph using scv.tl.velocity_graph. Differentiation macrostates, terminal states and absorption probabilities were inferred with CellRank (v1.5.1) using default parameters. Genes driving differentiation toward lining were identified by subsetting fibroblast and cartilage objects to trajectory-relevant clusters only (fibroblasts: synovial lining, F1, F2, F4; cartilage: CZ lining and C2). Identified genes are listed in Supplementary Table 9. To visualize key gene dynamics along the lining polarization trajectory (Fig. 3c), a generalized additive model was fitted to splicing trends along velocity pseudotime.

### Gene regulatory network analysis

To infer transcriptional networks regulating activation of the lining program, we used pySCENIC (v0.12.1) following the recommended workflow^21^. GRNBoost2 was applied to the gene expression matrix using ‘pyscenic grn’ with default parameters to infer gene regulatory networks. Human transcription factor and motif annotation databases were obtained from the Aerts Lab GitHub (https://github.com/aertslab). Resulting transcription factor–gene coexpression modules were pruned with RcisTarget using the ‘pyscenic ctx’ command with default settings to identify enriched motifs and predict regulons. This step retains modules (regulons) for which the regulator’s binding motif is enriched across the target genes. Regulon activity in individual cells was quantified with the ‘pyscenic aucell’ command (area under the curve threshold of 0.1), and the regulon matrix was binarized to assign active or inactive regulons by cell or cluster. Regulon cluster specificity was assessed using the regulon specificity score (RSS). Regulons predicted to govern lining differentiation were visualized in Cytoscape v3.10.1 with the iRegulon plugin.

### Gene set enrichment analysis (Progeny)

PROGENy (v1.16.0) was used to infer pathway activity in the embryonic synovium and cartilage^20^. We generated pseudobulk gene expression profiles of each cluster for every donor (*n* = 105), filtered to excluded genes with less than ten reads across samples. Counts were normalized using DESeq2. To determine pathway enrichment scores, we used Decoupler (v.1.5.0)^52^ to fit a multivariate linear model method for each pathway based on pathway–gene interaction weights. A positive pathway score indicates that the pathway is active.

### Projection of transcriptional signatures between adult and embryonic stromal cells

Adult LL and SL gene signatures were derived from published scRNAseq data from human RA synovium^14^. Raw counts were generated from FASTQ files using the CellHub pipeline (https://github.com/sansomlab/cellhub/) and Cell Ranger v7.0. Marker genes for the published LL and SL populations were identified using Seurat’s FindMarkers function. The top 100 markers for each population (Supplementary Table 15) were used to score cells in the embryonic stromal dataset with AddModuleScore (Seurat v5; control features = 5).

To map embryonic populations to adult fibroblasts from individuals with RA and OA, single-cell expression matrices from the AMP2 study^6^ were obtained from the authors. Homologous subpopulations across adult and embryonic fibroblasts were identified by Spearman correlation using the top 100 markers for each cluster (FindAllMarkers with Wilcoxon testing and average log_2_ (fold change) > 0.5). Module scores were calculated (with Seurat AddModuleScore as described above) for single-cell and pseudobulked adult stromal populations (aggregated by cluster and donor), using the top 100 markers derived from embryonic populations. The same module scores were generated using Visium spatial transcriptomic data from individuals with RA and OA, including early RA (*n* = 6), established RA (*n* = 11), resolving inflammatory arthritis (*n* = 5) and OA (*n* = 6).

### Flow cytometry

Dissociated cells were stained for surface markers by resuspending in 100 μl of PBS supplemented with 10% FBS containing antibodies at the appropriate dilution (Supplementary Table 25) and the live/dead dye 7-AAD (BD Biosciences). Samples were incubated with the antibody solution for 20 min at 4 °C. After washing with PBS, cells were fixed with fixation buffer for 20 min at room temperature, run on a BD LSRII or Fortessa and analyzed using FlowJo 10.8.1. All antibody details can be found in Supplementary Table 25.

### Immunofluorescence imaging

Whole joints were fixed in 10% neutral buffered formalin for 24–48 h. Samples older than 9 pcw were additionally decalcified in 10% EDTA before dehydration, paraffin embedding and sectioning at 5 µm. Slides were antigen retrieved and stained according to the ‘Antigen retrieval, blocking and staining protocol’ (Supplementary Information). Antibody details and concentrations are listed in Supplementary Table 25. Imaging was conducted using either a Zeiss 880 confocal microscope (tile-scans at ×20 magnification) or the GE CellDIVE system (×20 magnification), of which a detailed account is provided elsewhere^30^. Briefly, autofluorescence was recorded before antibody staining, samples were stained and reimaged, and background images were subtracted from stained images.

### Image analysis

To quantify the spatial distribution of PI16^+^ fibroblasts, fingers from *n* = 3 donors were serially sectioned and stained with antibodies to PI16, VWF and PRG4 (according to Supplementary Table 25). Sections were imaged on the CellDIVE system across four batches. The following methods were used to quantify PI16 staining and spatial distribution. Any sections where soft tissue surrounding the joint was damaged or contained strong staining artifacts were removed from further analysis.

#### Analysis with QuPath

DIP and PIP joint soft tissue regions were defined manually according to the protocol outlined in ‘Defining DIP and PIP soft tissue in QuPath’ (Supplementary Information). A list of analyzed images and raw data are provided in Supplementary Table 26. PI16^+^ area within each joint soft tissue region was quantified using pixel thresholds in Qupath (v0.6.0) for PI16 and DAPI (Supplementary Table 27). A separate threshold was applied to batch 1 because of reduced signal of PI16 staining in the 488 channel relative to the 647 channel used in other batches. To account for cellularity, PI16^+^ area was divided by DAPI^+^ area for each region. PI16:DAPI ratios were then normalized within each donor by dividing each value by the mean DIP value, and fold change relative to the DIP joint was plotted. Statistical analysis was performed in GraphPad Prism v10 using a paired *t*-test, with *P* < 0.05 considered significant.

#### Bespoke image analysis tool

Whole-cell and nuclear segmentation was performed using CellPose’s (v4.0.7) ‘cyto2’ and ‘nuclei’ models, respectively, in a modified script^24^. Cytoplasmic/cell surface markers (PRG4, PI16 and VWF) and a nuclear marker (DAPI) were aggregated for this purpose. The image scale was 1 pixel = 0.325 μm. Nuclear, cellular and cytoplasmic masks were generated, and nuclear masks were assigned to cellular regions to eliminate anuclear cells from analysis. Feature extraction was divided into morphological and intensity-based components. Morphological features extracted included area (cell, cytoplasm and nucleus), perimeter (cell and nucleus), eccentricity (cell and nucleus), solidity (cell and nucleus), extent (cell), minor axis (cell) and major axis (cell). Intensity of expression was extracted from cell boundaries, normalized to cellular area and scaled via inverse hyperbolic sine transformation. Cells were flagged for quality control parameters and filtered by area (5 ≤ *x* ≤ 4,000 μm) and mean absolute deviation of expression (±3 mean absolute deviation), and anucleate cells were removed. Tissue-level annotations were defined according to ‘Defining tissue annotations in bespoke spatial analysis tool’ (Supplementary Information). Cells were provided phenotype annotations via multiple methods. Per-image thresholding via triangle and otsu methods identified cells expressing high intensity for each stain. In parallel, each slide was integrated into a single object via Harmony and clustered via the Leiden algorithm (scanpy v1.11.5)^53^. These clusters were manually reviewed and annotated based on marker expression. Sections used for quantification of cell types are listed in Supplementary Table 28.

#### Spatial analysis with MuSpAn

Spatial analysis was performed in MuSpAn v1.2.1 (ref. ^25^). Point clouds denoting cell centroids were plotted using this package. The cross-PCF^25^ determines the significance of interactions between two cell types (for example, cells of type A and B) across defined length scales, with length scales in the range 0–100 μm being of biological significance. The *x* and *y* axes visualize the length scale, *r*, and the value of the PCF, *g*(*r*), respectively. Length scales *r* for which *g*(*r*) > 1 indicate clustering of type A cells around type B cells. In this study, the distribution of PI16^+^, VWF^+^ and PRG4^+^ populations was assessed with respect to each other to identify length scales on which these cells are more strongly cluster around each other than expected by chance. Sections used for generation of cross-PCF graphs (Fig. 5g) are listed in Supplementary Table 28. TCMs visualize colocalization and exclusion between cells of types A and B across a region of interest^25^. In this study, TCMs are visualized for each whole finger image depicting the association of PI16^+^ cells around VWF^+^ cells.

### RNAscope

Samples were fixed and paraffin embedded as previously described. To avoid decalcification, samples greater than 9 pcw were cut from either side of the joints to avoid developing bone. RNAscope was performed using the chromogenic singleplex (2.5 HD Reagent Kit-RED, ACD, 322360) and duplex (2.5 HD Duplex Assay, ACD, 322430), according to the manufacturer’s instructions. Images were acquired using an Olympus BX51 light microscope or a Zeiss Axioscan-7 slide scanner. Details regarding the RNAscope probes can be found in Supplementary Table 29.

### Generation of cell lines and in vitro culture

Joints were dissected and digested according to the ‘Embryonic joint processing’ with digestion protocol 3 (Supplementary Information). Single-cell suspensions were resuspended in DMEM + 4.5 g l^−1^ glucose and L-glutamine (11995-065) supplemented with penicillin/streptomycin and 10% FBS and cultured at 37 °C in 5% CO_2_. After 24 h, cultures were washed with PBS, and fresh medium was applied to remove nonadherent cells. Once cells reached 70% confluency, they were detached using TryPLE and passaged. For hypoxia experiments, cells were placed in a hypoxia chamber at 1% O_2_ for 72 h, with or without 100 ng ml^−1^ recombinant HBEGF (R&D, 259-HE-250/CF) and BTC (R&D, BT-BTC-050).

### Isolation and stimulation of CD34^+^PI16^+^ and CD34^−^PI16^−^ fibroblasts in vitro

Embryonic fibroblast populations were isolated from fingers of *n* = 3 independent donors (16–17 pcw). Joints were processed according to ‘Embryonic joint processing’ with digestion protocol 3 (Supplementary Information) with the following exceptions to maximize *PI16*^+^ fibroblasts. Skin was removed, leaving all tendon, ligaments, capsule and synovium intact. Cells from DIP and PIP joints were pooled once digested and stained according to the ‘Staining cells for FACS’ protocol (Supplementary Information). CD45^−^CD31^−^PDPN^+^ fibroblast populations were sorted into THY1^+^CD34^+^ and THY1^+^CD34^−^ populations using a FACSAria III (BD Biosciences). Isolated cells were cultured as previously described and plated for stimulation experiments at passage 1–2. Cells were stimulated with or without TNF (10 ng ml^−1^) and IL-1β (1 ng ml^−1^) for 24 h before lysis in RLT buffer (Qiagen) and RNA extraction (Qiagen micro-kit, 74004).

### Bulk sequencing and analysis

RNA samples were shipped to Novogene for library preparation and paired-end sequencing on an Illumina PE150 platform. Reads were processed using an in-house pipeline incorporating Trim Galore (v0.6.6 with default parameters, quality score of <20) for adapter trimming and quality control, STAR^54^ for genome alignment (v2.7.3a, spliced-alignment mode against the GRCh38 reference genome using Ensembl release 101) and featureCounts (v2.0.1) for read summarization and count generation. Genes with fewer than ten counts in fewer than four samples were excluded from downstream analysis.

#### GSVA

Top markers for the single-cell F2 population (adjusted *P* < 0.05, log_2_ (fold change) > 0.5) were used to create a gene signature characteristic of in vivo PI16^+^ fibroblasts. GSVA (v1.50.5) was used to assess enrichment of this signature in CD34^+^PI16^+^ and CD34^−^PI16^−^ in vitro fibroblasts. Normalized expression values (variance stabilized) were used as input for per-sample GSVA scoring.

#### Differential expression

Differential gene expression analysis was performed with DESeq2 (v1.42.1, normalization: size factor estimation, donor ID in all model designs). Genes with adjusted *P* values (Wald test, with the Benjamini–Hochberg method) of <0.05 and log_2_ (fold change) values of >0.5 were considered significant. The specific comparisons tested with model parameters are described in ‘Bulk RNA sequencing differential expression models’ (Supplementary Information). Supplementary Tables 16–20 contain differential gene expression results for each comparison.

#### Gene ontology enrichment

Gene Ontology over-representation analysis was performed with gsfisher (v0.2) on differentially expressed genes. Significant Gene Ontology biological process terms were defined as a Benjamini–Hochberg-adjusted *P* value of <0.05, with 10–500 genes per term and at least 5 foreground genes. To reduce redundancy, Gene Ontology terms were grouped by shared foreground genes using Louvain clustering (igraph v2.1.4, resolution 0.7) on a Jaccard similarity matrix. Small clusters were removed (less than ten terms for control comparisons and less than three terms for treatment comparisons), and each remaining cluster was assigned a general biological label (Supplementary Tables 21–24).

#### Fisher’s exact test

Genes characteristic of *PI16*^+^ populations, conserved in vivo and in vitro, were identified by intersecting differentially expressed genes from control in vitro (*CD34*^+^*PI16*^+^ compared to *CD34*^−^*PI16*^−^) and pseudobulked single-cell (F2; *PI16*^+^ compared to F1–6; *PI16*^−^) datasets. Fisher’s exact test (fisher.test, stats) was used to assess enrichment of this conserved gene set among genes significantly up- or downregulated by treatment in *CD34*^+^*PI16*^+^ and *CD34*^−^*PI16*^−^ fibroblasts.

### qPCR

Cells were lysed in RLT with 40 mM DTT, and RNA was extracted using a Qiagen micro-kit (74004), according to the manufacturer’s instructions. cDNA synthesis was performed using a First Strand Synthesis kit (Thermo Fisher, K1612) and diluted to a working concentration of 4 ng μl^−1^ in distilled water. For qPCR analysis, 5 ml of cDNA, 5.5 ml of Taqman-Fast master mix (Life Technologies, 4444963) and 0.5 ml of Taqman assay (Supplementary Table 29) for both the gene of interest and housekeeping control (*RPLP2*) were loaded into each well. Samples were run on a ViiA7 system (Thermo Fisher), and fold change in gene expression was calculated as ${2}^{-\varDelta \varDelta {C}_{{\rm{t}}}}$ relative to controls. Statistical analysis was performed in GraphPad Prism v9.

### Synchrotron X-ray tomography

#### In-line phase contrast synchrotron tomography

Samples were processed according to ‘Whole finger processing for synchrotron X-ray tomography’ (Supplementary Information) and imaged at the Diamond Manchester Imaging beamline, I13-2, at Diamond Light Source^55^ (beamtime: MG30542, MG34348). Images were obtained using a partially coherent, near-parallel, polychromatic ‘pink’ X-ray beam (27 keV) and a pco.edge 5.5 detector (PCO), coupled with an sCMOS sensor (2,560 × 2,160 pixels), LuAG.Ce scintillator and light microscope objectives. Both a ×2 objective (1.625-μm pixel size, 4.2 ×3.5 mm field of view) and a ×4 objective (0.8125-μm pixel size and 2.1 ×1.8 mm field of view) were used to collect images. Projection images were acquired at equally spaced angles over 180° of continuous rotation (‘fly scan’). Dark- and flat-field images were collected to normalize projections. The number of projections was chosen to give a good signal:noise ratio while minimizing any sample deformations that occurred during scan time. Exposure times, propagation distances and number of projections for each sample are listed in Supplementary Table 30.

#### Reconstruction and image processing

Image volumes were reconstructed using the Savu pipeline^56,57^, incorporating flat- and dark-field correction, optical distortion correction, ring artifact suppression, automatic rotation center calculation, radial distortion correction, zinger removal and ring artifact removal^58–62^. Paganin filtering^63^ was applied to projections before filtered back projection reconstruction, and parameters for each sample are listed in Supplementary Table 30. All image volumes were rescaled to 8 bit, and further processing was performed in Avizo3D (2021.1, Thermo Fisher Scientific). An unsharp mask filter (3D, edge size 5 pixels, edge contrast 3) was applied to sharpen small features, while a nonlocal means denoising filter (3D, spatial s.d. = 5, intensity s.d. = 0.2, search window 10 pixels, local neighborhood 3 pixels) with small local neighborhood was subsequently used to increase contrast and denoise images. Synovium, ligament and tendon structures were manually segmented every 25/50 slices. The interpolation tool was used to connect each segment, and 3D projections were created using the volren tool.

### Reporting summary

Further information on research design is available in the Nature Portfolio Reporting Summary linked to this article.

## Online content

Any methods, additional references, Nature Portfolio reporting summaries, source data, extended data, supplementary information, acknowledgements, peer review information; details of author contributions and competing interests; and statements of data and code availability are available at 10.1038/s41590-026-02542-2.

## Supplementary information

Supplementary Information Supplementary Methods.

Reporting Summary

Peer Review File

Supplementary Table 1 Sample overview.

Supplementary Table 2 List of scRNAseq embeddings.

Supplementary Table 3 Top markers for ‘all cells’ embedding (Extended Data Fig. 1b).

Supplementary Table 4 Top markers for ‘all cells main figure’ embedding (Fig. 1c).

Supplementary Table 5 Top markers for ‘stromal cells 1’ embedding (Extended Data Fig. 2a).

Supplementary Table 6 Top markers for ‘stromal cells 2’ embedding (Fig. 2a and Extended Data Fig. 2d).

Supplementary Table 7 Top markers for ‘STFs’ embedding (Fig. 3a).

Supplementary Table 8 Top markers for ‘cartilage cells’ embedding (Fig. 3b).

Supplementary Table 9 Genes driving lining differentiation identified by trajectory analysis (Fig. 3).

Supplementary Table 10 Numbers of tenocytes identified in each sample.

Supplementary Table 11 Literature sources for markers of extrasynovial joint structures.

Supplementary Table 12 Gene set over-representation analysis of STF and CZSC chondrocyte clusters (Extended Data Fig. 5).

Supplementary Table 13 Differentially expressed genes for F1 versus F2 (Extended Data Fig. 5).

Supplementary Table 14 Gene regulons (Extended Data Fig. 6).

Supplementary Table 15 Identified LL and SL top markers from RA (Fig. 2h).

Supplementary Table 16 Differentially expressed genes for in vitro *CD34*^−^*PI16*^−^ fibroblasts treatment versus control (Extended Data Fig. 9f).

Supplementary Table 17 Differentially expressed genes for in vitro *CD34*^+^*PI16*^+^ fibroblasts treatment versus control (Extended Data Fig. 9f).

Supplementary Table 18 Differentially expressed genes for in vitro *CD34*^+^*PI16*^+^ versus *CD34*^−^*PI16*^−^ fibroblasts under control conditions (Fig. 6b,c).

Supplementary Table 19 Differentially expressed genes for in vitro *CD34*^+^*PI16*^+^ versus *CD34*^−^*PI16*^−^ fibroblasts with an interaction term between treatment and cell type (Fig. 6f).

Supplementary Table 20 Differentially expressed genes for pseudobulked *PI16*^+^ (F2) *PI16*^−^ (F1–F6) fibroblasts from scRNAseq data (Extended Data Fig. 9c,d).

Supplementary Table 21 Gene Ontology biological processes based on differentially expressed genes downregulated to a greater extend in *CD34*^+^*PI16*^+^ fibroblasts, determined using an interaction term between treatment and cell type (Fig. 6f).

Supplementary Table 22 Clustered Gene Ontology biological processes based on differentially expressed genes upregulated to a greater extent in *CD34*^+^*PI16*^+^ fibroblasts, determined using an interaction term between treatment and cell type (Fig. 6f).

Supplementary Table 23 Clustered Gene Ontology biological processes based on differentially expressed genes from *CD34*^+^*PI16*^+^ versus *CD34*^−^*PI16*^−^ fibroblasts under control conditions (Fig. 6b).

Supplementary Table 24 Clustered Gene Ontology biological processes based on differentially expressed genes from pseudobulked *PI16*^+^ (F2) *PI16*^−^ (F1–F6) fibroblasts from scRNAseq data (Extended Data Fig. 9c).

Supplementary Table 25 Details of antibodies used in this manuscript.

Supplementary Table 26 Quantification of PI16^+^ and DAPI^+^ area determined by Qupath in serial finger sections (Fig. 5c).

Supplementary Table 27 Thresholds used for pixel classifiers to quantify PI16^+^ and DAPI^+^ area in by Qupath (Fig. 5c).

Supplementary Table 28 List of sections used for bespoke image analysis tool (Fig. 5 and Extended Data Fig. 8).

Supplementary Table 29 Details of RNAscope and Taqman probes used in this manuscript.

Supplementary Table 30 Details of parameters used for acquiring and processing X-ray tomography images.

Supplementary Table 31 List of publicly available data and their sources used in this manuscript.

## Data Availability

For the embryonic sequencing data, scRNAseq FASTQ files and H5AD objects are available on Array Express (E-MTAB-14722). For bulk RNA-seq data of in vitro fibroblasts, FASQ files and raw counts can be found on Gene Expression Omnibus (GSE328951). For adult disease data, the adult data sets, the figures they are featured in and the sources from which they were obtained are listed in Supplementary Table 31. OA and RA data from the AMP2 consortium^6^ were obtained from https://www.synapse.org/Synapse:syn52420382 with approval from the contributors. RA data from Wei et al.^14^ were obtained from https://www.immport.org under code SDY1599. Visium spatial transcriptomics data of OA and RA synovium are described in Reis Nisa et al.^64^. The cross-tissue fibroblast single-cell atlas from Korsunsky et al.^30^ was obtained from the Broad Single Cell Portal under accession number SCP738. Input images for our bespoke spatial analysis tool and the processed objects generated can be found at https://www.ebi.ac.uk/biostudies/bioimages/studies/S-BIAD3270.

## Code Availability

Documentation and code for our bespoke spatial analysis tool can be found at GitHub at https://github.com/mjbcowan/2026_NI_Manuscript.
