# Supplementary material for: The embryonic origins of site-specific arthritis
Source: Nat Immunol. 2026 Jun 8;27(7):1390–403. doi: 10.1038/s41590-026-02542-2 (PMC13310765; doi:10.1038/s41590-026-02542-2)
Supplement: Supplementary file 1 — Supplementary Methods. [file 41590_2026_2542_MOESM1_ESM.pdf]

nature immunology

Supplementary information https://doi.org/10.1038/s41590-026-02542-2

The embryonic origins of site-specific arthritis

In the format provided by the authors and unedited

Supplementary methods

Integration, clustering and annotation of scRNAseq data

After excluding myeloid, endothelial, pericyte, glial, and tenocyte populations from the cross-lineage atlas, we focused on stromal cells. Inspection of the initial stromal embedding ("Stromal Cell 1", Fig. S3a) identified clusters likely representing extra-articular populations (cutaneous precursors: MSX1+RSPO4+ and TWIST2+, and tendon precursors: $TNMD^{high}$ SCX^{high}), which were removed from further analysis. Two low-quality clusters, characterised by MALAT1 or FOS/JUNB expression, were also excluded. The top 3,000 highly variable genes (HVGs) per sample were identified using scanpy.pp.highly_variable_genes (batch_key='sample_id', flavour='seurat_v3'), and their union was retained. Samples were integrated with scVI (batch_key='sample_id', n_latent=30), including cell-cycle gene expression as a continuous covariate. Post-integration, an exact neighbour graph was computed with the hnsw algorithm (n=20 neighbours, Euclidean distance) and used for UMAP and Leiden clustering.

Clusters were manually annotated and were further subclustered where heterogeneity was evident or merged when separation appeared technical rather than biological. In the "Stromal Cell 2" layout (Fig. 2a), we identified STF (ZFHX4+), CZSC (CLU+), and chondrocytes (COL9A2+). STFs were separated from CZSCs and chondrocytes and both subsets were re-analysed using the same workflow, including HVG rediscovery, integration, UMAP calculation, and Leiden clustering. Further details are provided in Supplementary Table 2, and cluster markers for all embeddings are listed in Supplementary Tables 3–8.

Embryonic joint processing

Skin and tendon material was removed as much as possible from each finger. However, we noticed that a small number of contaminating tenocytes were present in 3 out of 7 PIP samples (n=32, n=79, n=4 cells; Supplementary Table 10). DIP and PIP joints were isolated by cutting either side of the joint space, cut into pieces and enzymatically digested (digestion protocol2: 1mg/ml collagenase D or digestion protocol 3: 0.1mg/ml of liberaseTL) in DMEM, at 37C for 1h 30min. The digestion protocol used for each sample is recorded supplementary table 1. In both protocols, supernatant containing released cells was collected every 10-15min, neutralized in DMEM 10% FBS, and stored at 4C. Once supernatant was removed, fresh enzyme was applied to continue sample digestion. After digestion, the remaining tissue was manually dissociated, combined with collected supernatants and passed through a 70μm filter.

Whole finger processing for synchrotron X-ray tomography

Whole fingers were fixed in 10% neutral buffered formalin for 24-72hs, depending on the sample size. Samples were dehydrated, incubated in Xylene (2x 1h) and embedded in paraffin, using standard histology processing. Wax was melted to leave ~1mm of wax around each finger.

Antigen retrieval, blocking and staining protocol

Formalin-fixed paraffin-embedded (FFPE) slides were permeabilised in 0.3% Triton X-100 for 10min and washed in PBS for 5min. Antigen retrieval was performed in an NxGen

decloaking chamber (Biocare Medical) using citrate buffer (Agilent, S1699; pH 6, 20min) and Tris-based buffer (pH9, 20min). Slides were then blocked for 1h at room temperature (RT) in blocking buffer; 3% BSA, 10% donkey serum, and human FcR Blocking Reagent (Miltenyi, 130-059-901; 1:200) in PBS. After washing in PBS, slides were stained with DAPI (Thermo, D3571) for 15min, washed again, coverslipped in mounting medium (50% glycerol -Sigma, G5516 and 4% propyl gallate – Sigma, 2370), and stored at 4C. Slides were de-coverslipped in PBS and incubated with primary antibodies in blocking buffer (supplementary methods) overnight at 4C. After three washes in PBS with 0.05% Tween-20, secondary antibodies were applied for 1h at RT where required.

Staining cells for FACS

Once single cell suspensions were collected, cells were incubated with FC block (1:100) and a live/dead stain (Near-IR fluorescent reactive dye, 1:1000, Invitrogen, L10119A) in PBS for 20 min at RT. After washing with PBS, cells were stained with antibodies in PBS 1% BSA, according to Supplementary table 25, for 20min at 4C and washed (PBS 1% BSA), before filtering through 70μm strainers.

Defining DIP and PIP soft tissue in QuPath

The DIP region was defined from the midpoint between the PIP and DIP joint spaces to 2000 μm distal to the DIP joint space, or to the fingertip if this distance was less than 2000 μm. The PIP region was defined from the midpoint between the PIP and DIP joint spaces to 2000 μm proximal to the PIP joint space, or to the base of the finger if this distance was

less than 2000 μm. When the MCP joint was present, the PIP region extended from the midpoint between the DIP and PIP joints to the midpoint between the PIP and MCP joints (Extended Data Fig. 8). Within these regions, joint soft tissue was manually annotated using the wand tool, including tendon, ligament, and synovium, but excluding cartilage and skin.

Defining tissue annotations in bespoke spatial analysis tool

Each OME-TIFF was annotated in QuPath (v0.6.0) to identify histological structures such as skin epithelium ("'UBERON:0019204'), dense regular connective tissue ("UBERON:0007845"), bone ("UBERON:0002481"), loose connective tissue ("UBERON:0007844") and also artefactual regions of tissue damage (which were excluded). Further annotations such as such as "distal" and "proximal" regions of interest were annotated in QuPath by bisecting the middle phalanx and subsequently integrated into the spatialData object. Dense regular connective tissue was assessed by manual annotation and related to regions resembling ligaments or tendons of high cellular density within the images.

Bulk RNA sequencing differential expression models

DE for CD34+Pi16+ compared to CD34-Pi16- fibroblasts under control conditions:

DE was performed on control samples only, with fibroblast sample ID and cell type included in the model (Supplementary Table 18).

DE for PI16+ fibroblasts compared to PI16- fibroblasts from scRNAseq:

F2 *PI16*+ and *PI16*- fibroblasts (F1-F6) from the scRNA-seq data were pseudobulked in Seurat (v5). DE was performed with sample ID and cell type included in the model (Supplementary Table 20).

Identifying 'treatment responsive' DE genes with a likelihood ratio test (LRT):

Likelihood ratio tests (LRTs) compared a full model including treatment, with a reduced model excluding treatment. Genes with BH-adjusted p-value < 0.05 were considered treatment responsive. Hierarchical clustering (hclust, stats v4.3.2) and PCA (prcomp) were performed on scaled VST expression values to assess sample similarity and separation by cell type and condition. Overlap between up and downregulated 'treatment-responsive genes' in each fibroblast population was visualised using Venn diagrams (eulerr v7.0.4).

DE genes for TNF and IL1β treatment compared to control in CD34+Pi16+ or CD34- Pi16- fibroblasts:

Separate models were run for each cell type with sample ID and treatment included as terms (Supplementary Tables 16–17).

Genes that respond differently to TNF and IL1β depending on cell type:

To test whether TNF and IL1β responses differed between fibroblast populations, an interaction term between cell type and treatment was added to the design formula (Supplementary Table 19). Genes showing stronger treatment responses in each cell type were identified by intersecting significant interaction-model genes with genes up or downregulated by treatment in that cell type. For example, to identify genes that are more upregulated with treatment in CD34+Pi16+ cells, genes from the interaction model were

intersected with genes upregulated in CD34+Pi16+ fibroblasts without an interaction term.
