# Supplementary material for: The embryonic origins of site-specific arthritis
Source: Nat Immunol. 2026 Jun 8;27(7):1390–403. doi: 10.1038/s41590-026-02542-2 (PMC13310765; doi:10.1038/s41590-026-02542-2)
Supplement: Supplementary file 2 — Reporting Summary [file 41590_2026_2542_MOESM2_ESM.pdf]

Corresponding author(s): Stephen Sansom, Mark C Coles Christopher D Buckley

Last updated by author(s): Apr 24, 2026

Reporting Summary

Nature Portfolio wishes to improve the reproducibility of the work that we publish. This form provides structure for consistency and transparency in reporting. For further information on Nature Portfolio policies, see our Editorial Policies and the Editorial Policy Checklist.

Statistics

For all statistical analyses, confirm that the following items are present in the figure legend, table legend, main text, or Methods section.

n/a	Confirmed	
☐	☒	The exact sample size (*n*) for each experimental group/condition, given as a discrete number and unit of measurement
☐	☒	A statement on whether measurements were taken from distinct samples or whether the same sample was measured repeatedly
☐	☒	The statistical test(s) used AND whether they are one- or two-sided<br>*Only common tests should be described solely by name; describe more complex techniques in the Methods section.*
☐	☒	A description of all covariates tested
☐	☒	A description of any assumptions or corrections, such as tests of normality and adjustment for multiple comparisons
☐	☒	A full description of the statistical parameters including central tendency (e.g. means) or other basic estimates (e.g. regression coefficient) AND variation (e.g. standard deviation) or associated estimates of uncertainty (e.g. confidence intervals)
☐	☒	For null hypothesis testing, the test statistic (e.g. *F*, *t*, *r*) with confidence intervals, effect sizes, degrees of freedom and *P* value noted<br>*Give P values as exact values whenever suitable.*
☒	☐	For Bayesian analysis, information on the choice of priors and Markov chain Monte Carlo settings
☒	☐	For hierarchical and complex designs, identification of the appropriate level for tests and full reporting of outcomes
☐	☒	Estimates of effect sizes (e.g. Cohen's *d*, Pearson's *r*), indicating how they were calculated

Our web collection on statistics for biologists contains articles on many of the points above.

Software and code

Policy information about availability of computer code

Data collection	Documentation and code and for our bespoke spatial analysis tool can be found here: https://github.com/mjbcowan/2026_NI_Manuscript
Data analysis	This data was analyzed used standard packages developed in python or R, package versions are stated in the 'Methods' section of the manuscript and below:<br>Cell Ranger 4.0.0, 7.0 - version used for each analysis stated in manuscript<br>Seurat 4.3 or 5 - version used for each analysis stated in manuscript<br>scrublet 0.2.3<br>SIngleCellExperiment 1.14.1<br>tenxutils 0.1<br>ggplot2 3.4.2<br>scVI 0.12.09<br>scVelo 0.2.5<br>CellRank 1.5.1<br>pySCENIC 0.12.1<br>Cytoscape 3.10.1<br>PROGENy 1.16.0<br>Decoupler 1.5.0<br>Qupath 0.6.0<br>CellPose 4.0.7<br>ScanPy 1.11.5<br>MuSpAn 1.2.1

Trim Galore 0.6.6
STAR 2.7.3
featureCounts 2.0.1
GSVA 1.50.5
DEseq2 1.42.1
Stats 4.3.2
eulerr 7.0.4
gsfisher 0.2
igraph 2.1.4
GraphPad v9 and 10- version used for each analysis stated in manuscript
Avizo 2021.1

For manuscripts utilizing custom algorithms or software that are central to the research but not yet described in published literature, software must be made available to editors and reviewers. We strongly encourage code deposition in a community repository (e.g. GitHub). See the Nature Portfolio guidelines for submitting code & software for further information.

Data

Policy information about availability of data

All manuscripts must include a data availability statement. This statement should provide the following information, where applicable:

- Accession codes, unique identifiers, or web links for publicly available datasets
- A description of any restrictions on data availability
- For clinical datasets or third party data, please ensure that the statement adheres to our policy

Data availability
Embryonic sequencing data:
scRNAseq FASTQ files and H5AD objects are available on Array Express (E-MATB-14722). Bulk RNA sequencing data of in vitro fibroblasts:
FASQ files and raw counts can be found on GEO (GSE328951).
Adult disease:
Adult data sets the figures they feature in, and the sources from which they were obtained are listed in Supplementary Table 31. OA and RA data from AMP2 consortium6 was obtained from (https://www.synapse.org/Synapse:syn52420382) with approval from the contributors. RA data from Wei et al 202014 was obtained from https://www.immport.org, code SDY1599. Visium spatial transcriptomics of OA and RA synovium was kindly provided by Prof. Adam Croft and Dr. Chris Mahony (University of Birmingham) and is described in the following pre-press article on bioarchive here: https://doi.org/10.1101/2024.09.20.614064. The cross-tissue fibroblast single-cell atlas from Korsunsky et al. was obtained from the Broad Single Cell Portal, with accession number SCP738.
Input Images for our bespoke spatial analysis tool and the processed objects generated can be found here: https://www.ebi.ac.uk/biostudies/bioimages/studies/S-BIAD3270

Research involving human participants, their data, or biological material

Policy information about studies with human participants or human data. See also policy information about sex, gender (identity/presentation), and sexual orientation and race, ethnicity and racism.

Reporting on sex and gender	The single cell sequencing atlas of human feotal finger joints was generated from 7 individuals, of which 6 were of female sex, and 1 was of male sex. Information on the sex of all samples is provided in the meta data. Samples were obtained from the human developmental biology resource (https://www.hdbr.org/). Upon initial collection information on sex is not available, and is provided at later date. Thus, for single cell sequencing it was not possible to equally represent both sexes.
Reporting on race, ethnicity, or other socially relevant groupings	Information on race and ethnicity is not provided.
Population characteristics	All samples included in single cell, imaging and x-ray tomography data have no chromosomal abnormalities. The single cell atlas was generated using both early (8-9 post conceptional weeks, pcw) and late (12-14 pcw) samples. To account for differences across developmental time, all analysis was performed on these groups separately as well as the dataset as a whole.
Recruitment	n/a
Ethics oversight	All feotal samples were provided by the human developmental biology resource (https://www.hdbr.org/), under the following RECs: 18/NE/0290 and 18/NE/0290 (Newcastle), 18/LO/0822 and 23/LO/0312 (UCL).

Note that full information on the approval of the study protocol must also be provided in the manuscript.

Field-specific reporting

Please select the one below that is the best fit for your research. If you are not sure, read the appropriate sections before making your selection.

☒ Life sciences ☐ Behavioural & social sciences ☐ Ecological, evolutionary & environmental sciences

For a reference copy of the document with all sections, see nature.com/documents/nr-reporting-summary-flat.pdf

Life sciences study design

All studies must disclose on these points even when the disclosure is negative.

Sample size	No statistical method was used to predetermine sample size. Sample sizes for human foetal tissue sequencing experiments were determined by the availability of rare human material and feasibility of data generation. Overall n=3 donors and n=4 donors were included for each developmental stage (early and late respectively. This sample size captured 65,705 cells, which was sufficient to identify reproducible cellular populations, spatial patterns, and joint-specific differences across independent donors. This provided enough power for single cell analysis and relevant statical tests were chosen for this sample size.<br><br>For other assays, sample sizes were chosen based on sample availability and standard practice in the field, with experiments typically performed using at least three independent biological replicates where feasible. The sample sizes used were sufficient to detect reproducible differences across conditions.<br><br>Specificall, for in vitro experiments, image quantification and spatial statistics, n=3 donors was used, which generated statically meaningful data. For Synchrotron X-ray tomography, synovial volumes were segmented from n=3 fingers obtained from n=2 donors, at 14-15pcw. Segmentation of all soft tissue structures was performed on n=1 donor 14-15pcw and n=1 donor 19pcw. Synchrotron beam time must be applied for 6months in advance, through a peer reviewed process, and 2-4 days is typically allocated. Thus, there is a limit on the number of samples that can be imaged. As such we have only quantified synovial volumes.
Data exclusions	For Qupath and MuSPAN quantification of Pi16 across serial finger sections, those with significant damage were excluded.
Replication	With the exception of some segmentation from synchrotron X-ray tomography images, all images were images on at least 2 independent donors and at least 3 if quantification was applied.All in vitro experiments were performed on 3 independent donors and results were reproduced across all donors. Single cell and in vitro experiments were used together to verify findings.
Randomization	Our main comparison was between PIP and DIP joints, which were mostly paired from the same donor, rather than treatment groups. Thus, randomization is not applicable in this instance.
Blinding	Similar to above, we are not comparing treatment groups but the anatomy and cellular composition of different joints. Thus blinding is less relevant to our study. However, statical tests were implemented to qualify findings.

Reporting for specific materials, systems and methods

We require information from authors about some types of materials, experimental systems and methods used in many studies. Here, indicate whether each material, system or method listed is relevant to your study. If you are not sure if a list item applies to your research, read the appropriate section before selecting a response.

Materials & experimental systems

n/a	Involved in the study	
☐	☒	Antibodies
☐	☒	Eukaryotic cell lines
☒	☐	Palaeontology and archaeology
☒	☐	Animals and other organisms
☒	☐	Clinical data
☒	☐	Dual use research of concern
☒	☐	Plants

Methods

n/a	Involved in the study	
☒	☐	ChIP-seq
☐	☒	Flow cytometry
☒	☐	MRI-based neuroimaging

Antibodies

Antibodies used	The following antibodies, company, catalogue number and lot number, used for immunofluorecent imaging are provided. COL1A1 (Novus, cat: NBP1-30054, lot: SH6220) COL9A2 (Atlas, cat: HPA056316, lot: R74939), TPPP3 (abcam, cat: ab150998, lot: GR3301108-6), CA9 (R&D, cat: AF2188, lot: VNQ022009A), CD31 (647 conjugated, Novus, cat : BP2-34578AF647, lot: D139176), Pi16 (R&D, cat: AF4980, lot: CBEB0222101), PRG4 (Merk, cat: MABT401, lot: 3745921), VWF (abcam, cat: ab9378).<br><br>The following antibodies, clone and catalogue number, used for flow cytometry and FACs sorting are provided: CD31 AF700 (Biolegend, WM59, cat: 303133), CD31 FITC (Biolegend, WM59, cat: 303104), CD45 FITC (Biolegend, H130, cat: 304006), CD45 BV785 (Biolegend, H130, cat: 304048), CD34 647 (Biolegend, 581, cat: 343507), Thy1 PECy7 (Biolegend, 5E10, cat: 328124), PDPN PE (Biolegend, NC-08, 337003).
Validation	Immunofluorescence:<br>COL1A1: https://www.rndsystems.com/products/collagen-i-alpha-1-antibody_nbp1-30054<br>COL9A2: https://www.atlasantibodies.com/products/primary-antibodies/triple-a-polyclonals/anti-col9a2-antibody-hpa056316/<br>TPPP3: https://www.abcam.com/en-us/products/primary-antibodies/tppp3-antibody-ab150998<br>CA9: https://www.rndsystems.com/products/human-carbonic-anhydrase-ix-ca9-antibody_af2188<br>CD31: https://www.novusbio.com/products/cd31-pecam-1-antibody-c313-jc-70a_nbp2-34578af647?srsltid=AfmBOorTt9QX5HhtBMbjBZdw6oADsVeOgOYN3Y5-BmEBFWI2aSa0bMA9

Pi16: https://www.rndsystems.com/products/human-peptidase-inhibitor-16-pi16-antibody_af4980?gclsrc=aw.ds&gad_source=1&gad_campaignid=20161432922&gbraid=0AAAAAD_kmX1t8ajaIX6gtlsq8_W1Q9Cug&gclid=EAIaIQobChMIh_HHr_L_kwMV-5tQBh1k8RAuEAAYASAAEgLjU_D_BwE
PRG4: https://www.sigmaaldrich.com/GB/en/product/mm/mabt401
VWF: https://www.abcam.com/en-us/products/primary-antibodies/von-willebrand-factor-antibody-ab9378

Flow cytometry antibodies:
CD31 AF700: https://www.biolegend.com/ja-jp/products/alexa-fluor-700-anti-human-cd31-antibody-12380
CD31 FITC: https://www.biolegend.com/en-ie/products/fitc-anti-human-cd31-antibody-881?GroupID=BLG10311
CD45 FITC: https://www.biolegend.com/en-gb/products/fitc-anti-human-cd45-antibody-707
CD45 BV785: https://www.biolegend.com/en-ie/products/brilliant-violet-785-anti-human-cd45-antibody-9325
CD34 647: https://www.biolegend.com/en-ie/products/alexa-fluor-647-anti-human-cd34-antibody-6089
Thy1 PECY7: https://www.biolegend.com/en-ie/products/pe-cyanine7-anti-human-cd90-thy1-antibody-8282
PDPN PE: https://www.biolegend.com/en-ie/products/pe-anti-human-podoplanin-antibody-5798

Eukaryotic cell lines

Policy information about cell lines and Sex and Gender in Research

Cell line source(s): Primary fibroblast cultures were generated from embryonic synovial joints collected from hdbr (as previously described) that were dissected and processed as described in the 'Methods' section. Cells were cultured for a limited number of passages (below P10) to ensure in vivo features were retained.

Authentication: To ensure these cultures contain fibroblasts, the morphology of initial cultures generated was examined under the microscope and flow cytometry was performed on markers such as PDPN, CD31 and CD45, to check immune or endothelial cells were not present. However, flow cytometry may not have been performed on the lines specially used in this study.

Mycoplasma contamination: Myocoplamsa tests were performed regularly in the lab but we cannot confirm whether the cultures used in this manuscript were checked specifically.

Commonly misidentified lines (See ICLAC register): n/a

Plants

Seed stocks: *Report on the source of all seed stocks or other plant material used. If applicable, state the seed stock centre and catalogue number. If plant specimens were collected from the field, describe the collection location, date and sampling procedures.*

Novel plant genotypes: *Describe the methods by which all novel plant genotypes were produced. This includes those generated by transgenic approaches, gene editing, chemical/radiation-based mutagenesis and hybridization. For transgenic lines, describe the transformation method, the number of independent lines analyzed and the generation upon which experiments were performed. For gene-edited lines, describe the editor used, the endogenous sequence targeted for editing, the targeting guide RNA sequence (if applicable) and how the editor was applied.*

Authentication: *Describe any authentication procedures for each seed stock used or novel genotype generated. Describe any experiments used to assess the effect of a mutation and, where applicable, how potential secondary effects (e.g. second site T-DNA insertions, mosiacism, off-target gene editing) were examined.*

Flow Cytometry

Plots

Confirm that:

☒ The axis labels state the marker and fluorochrome used (e.g. CD4-FITC).

☒ The axis scales are clearly visible. Include numbers along axes only for bottom left plot of group (a 'group' is an analysis of identical markers).

☒ All plots are contour plots with outliers or pseudocolor plots.

☒ A numerical value for number of cells or percentage (with statistics) is provided.

Methodology

Sample preparation: Flow cytometry
To perform flow cytometry on feotal joints, skin and tendon material were first removed from each finger, and DIP and PIP joints isolated by cutting either side of the joint space. Joints were cut into pieces and enzymatically digested with 0.1mg/ml of liberaseTL in DMEM, at 37oC for 1h 30mins. Supernatant containing released cells was collected every 10-15 mins, neutralized in DMEM 10% FBS, and stored at 4oC. Once supernatant was removed, fresh enzyme was applied to continue sample digestion. After digestion, the remaining tissue was manually dissociated, and combined with collected supernatants, in DMEM 10% FBS. All isolated cells were then passed through a 70um filter.

Dissociated cells were stained for surface markers by resuspending in 100ul of PBS 10% FBS containing antibodies at the appropriate dilution and the live dead dye 7AAD (BD Biosciences). Samples were incubated with the antibody solution for

20mins at 4°C. Following washing with PBS, cells were fixed with fixation buffer, for 20mins RT, run on the BD LSRII or Fortessa X-20, and analyzed using FlowJo 10.8.1.

FACs sorting
Embryonic fibroblast populations were isolated from fingers of n=3 independent donors (16-17pcw). Skin was removed, leaving all joint soft tissue intact (tendon, ligaments, capsule and synovium). DIP and PIP joints were excised and pooled, followed by enzymatic digestion and filtering as previously described. Once single cell suspensions were collected, cells were incubated with FC block (1:100) and Near-IR fluorescent reactive dye (1:1000, Invitrogen, L10119A), which distinguishes dead cells, in PBS for 20 mins at RT. After washing with PBS, cells were stained with antibodies CD31 (WM59), CD45 (HI30), PDPN (NC-08), Thy1 (5E10), CD34 (581) at 1:200 in PBS 1% BSA. Samples were incubated with the antibody solution for 20mins at 4°C and washed in PBS 1% BSA, before further filtering through 70um cell strainers. CD45-CD31-PDPN+ fibroblast populations were sorted into Thy1+CD34+ and Thy1+CD34- using the FACs Aria III (BD bioscience) and collected in neat FBS to preserve cell viability.

Instrument	LSRII or Fortessa X-20, FACs Aria III (BD bioscience)
Software	FlowJo 10.8.1.
Cell population abundance	Flow cytometry was used to validate the frequencies of endothelial (CD31+, 1.05%), immune (CD45+, 1.4%) and stromal cells (CD31- CD45-, 96.5%) at 14-15pcw embryonic joints. This is reported in the manuscript
Gating strategy	Flow cytometry<br>Flow cytometry was used to validate the proportions of stromal (CD45- CD31-), immune (CD45+) and endothelial (CD31+) cells in feotal joints. An initial SSC and FSC gate was set to remove debris, which could be distinguished by low values. Single cells were then defined using FSCA and FSCH. Any clear doublets were not included. Alive cells were determined using the live/dead dye 7AAD. Within the "alive" fraction, the proportion of CD31+, CD45+, and CD31- CD45- cells was determined by comparing CD31 and CD45 values. Clear positive and negative populations were observed and gated accordingly.<br><br>FACs sorting<br>To identify cells for sorting an initial SSC and FSC gate was set to remove debris and single cells were then defined using FSCW and FSCH. Alive cells were determined using the live/dead dye Near-IR fluorescent reactive dye (1:1000, Invitrogen, L10119A). CD45-CD31-PDPN+ fibroblast populations were sorted into Thy1+CD34+ and Thy1+CD34- cells for culture.

☒ Tick this box to confirm that a figure exemplifying the gating strategy is provided in the Supplementary Information.
