# Supplementary material for: The embryonic origins of site-specific arthritis
Source: Nat Immunol. 2026 Jun 8;27(7):1390–403. doi: 10.1038/s41590-026-02542-2 (PMC13310765; doi:10.1038/s41590-026-02542-2)
Supplement: Supplementary file 3 — Peer Review File [file 41590_2026_2542_MOESM3_ESM.pdf]

nature portfolio

Peer Review File

The embryonic origins of site-specific arthritis

Corresponding Author: Professor Christopher Buckley

This file contains all editorial decision letters in order by version, followed by all author rebuttals in order by version.

Decision Letter:

3rd Jan 2025

Dear Professor Buckley,

Your Article, "The embryonic origins of site-specific arthritis" has now been seen by 2 referees. You will see from their comments copied below that while they find your work of considerable potential interest, they have raised quite substantial concerns that must be addressed. In light of these comments, we cannot accept the manuscript for publication, but would be very interested in considering a revised version that addresses these serious concerns.

We hope you will find the referees' comments useful as you decide how to proceed. If you wish to submit a substantially revised manuscript, please bear in mind that we will be reluctant to approach the referees again in the absence of major revisions. In particular, please provide some analysis of the function of PI16+ and ALDH1A1+ cells. Please do not hesitate to get in touch if you would like to discuss these issues further.

If you choose to revise your manuscript taking into account all reviewer and editor comments, please highlight all changes in the manuscript text file [OPTIONAL: in Microsoft Word format].

We are committed to providing a fair and constructive peer-review process. Do not hesitate to contact us if there are specific requests from the reviewers that you believe are technically impossible or unlikely to yield a meaningful outcome.

If revising your manuscript:

* Include a “Response to referees” document detailing, point-by-point, how you addressed each referee comment. If no action was taken to address a point, you must provide a compelling argument. This response will be sent back to the referees along with the revised manuscript.

* If you have not done so already please begin to revise your manuscript so that it conforms to our Article format instructions at http://www.nature.com/ni/authors/index.html. Refer also to any guidelines provided in this letter.

* Include a revised version of any required reporting checklist. It will be available to referees (and, potentially, statisticians) to aid in their evaluation if the manuscript goes back for peer review. A revised checklist is essential for re-review of the paper.

The Reporting Summary can be found here:
https://www.nature.com/documents/nr-reporting-summary.pdf

When submitting the revised version of your manuscript, please pay close attention to our href="https://www.nature.com/nature-portfolio/editorial-policies/image-integrity">Digital Image Integrity Guidelines. and to the following points below:

-- that unprocessed scans are clearly labelled and match the gels and western blots presented in figures.
-- that control panels for gels and western blots are appropriately described as loading on sample processing controls
-- all images in the paper are checked for duplication of panels and for splicing of gel lanes.

Extended Data figures and tables are online-only (appearing in the online PDF and full-text HTML version of the paper), peer-reviewed display items that provide essential background to the Article but are not included in the printed version of the paper due to space constraints or being of interest only to a few specialists. A maximum of ten Extended Data display items (figures and tables) is typically permitted. When re-submitting your manuscript, please ensure that any supplementary

figures and tables that are more critical to the manuscript’s conclusions are converted to Extended data to increase these data’s visibility.

Finally, please ensure that you retain unprocessed data and metadata files after publication, ideally archiving data in perpetuity, as these may be requested during the peer review and production process or after publication if any issues arise.

You may use the link below to submit your revised manuscript and related files:

Link Redacted

Note: This URL links to your confidential home page and associated information about manuscripts you may have submitted, or that you are reviewing for us. If you wish to forward this email to co-authors, please delete the link to your homepage.

If you wish to submit a suitably revised manuscript we would hope to receive it within 6 months. If you cannot send it within this time, please let us know. We will be happy to consider your revision so long as nothing similar has been accepted for publication at Nature Immunology or published elsewhere.

Nature Immunology is committed to improving transparency in authorship. As part of our efforts in this direction, we are now requesting that all authors identified as ‘corresponding author’ on published papers create and link their Open Researcher and Contributor Identifier (ORCID) with their account on the Manuscript Tracking System (MTS), prior to acceptance. ORCID helps the scientific community achieve unambiguous attribution of all scholarly contributions. You can create and link your ORCID from the home page of the MTS by clicking on ‘Modify my Springer Nature account’. For more information please visit please visit www.springernature.com/orcid.

Please do not hesitate to contact me if you have any questions or would like to discuss the required revisions further.

Thank you for the opportunity to review your work.

Sincerely,

Stephanie Houston, PhD
Senior Editor
Nature Immunology

Version 0:

Reviewer comments:

Reviewer #1

(Remarks to the Author)
The authors investigate site-specific inflammation in the distal interphalangeal (DIP) and proximal interphalangeal (PIP) joints using single-cell RNA sequencing (scRNAseq) and high-resolution X-ray tomography. They identify specific fibroblast populations, particularly Pi16+ fibroblasts, that may predispose these joints to inflammatory arthritis. This manuscript uniquely examines the cellular differences between DIP and PIP joints during fetal development and provides insights into how these fibroblast populations may influence arthritis susceptibility. It highlights the importance of developmental factors in anatomical predisposition.
The data and methodology are robust, with effective quality control measures enhancing the reliability of the dataset. The combination of scRNAseq and high-resolution imaging illustrates structural differences, making the presentation clear.
The study uses robust statistical techniques that control for multi-donor variability. Quality control measures strengthen conclusions about stromal cell predominance, effectively addressing uncertainties. Multi-donor analyses and complementary validation increase the reliability and robustness of the findings, thereby advancing the understanding of joint development and disease susceptibility. The abstract clearly summarises the study, while the introduction and conclusion effectively contextualise the research and its implications, enhancing accessibility and impact within the field.
Overall, this is an interesting paper. The paper is well written and the experiments are well conducted. However, additional work is required to improve the significance and impact of the findings, provided in a revised version.
-The article would benefit from a brief contextualisation of what is known about joint development.
-The authors should comment on why they detect a significant amount of tenocytes in their dataset, even though tendons were removed according to their descriptions in the Materials and Methods section. Is this due to contamination? Is the entheseal part also removed?
-Subgroups of fibroblasts were categorised according to their anatomical location. The authors also included functional categorisation, e.g. chondrocyte subsets, but this was primarily done by their anatomical localisation. Authors should provide co-expressing markers that are functionally related to the respective subsets of fibroblasts.
-ZFHX4 was assigned to tendons, but the authors excluded tendons from their data sets. Does this mean that tendons develop from STFs? CRABP1 is attributed to extra-synovial tissue. This needs further evidence. TPPP3 is attributed to articular soft tissue. Does this subtype of fibroblast develop sublining structures of the synovium?

-It is not clear which cells develop into capsule, which remain at the synovial-cartilage interface and which develop into extra-articular enthesis and tendon. The authors should provide more data on this.
-What is the relationship between ZFHX4 expression and CRABP1+ fibroblasts? It is unclear how the authors concluded that certain types of CRABP1+ cells develop into tendon and ligament populations when extra-articular cells were not included in the analysis. The authors should include extra-articular tissue datasets and re-analyse their conclusions.
-Functional differences between tendon-related subtypes of fibroblasts and PI16+ and ALDH1A1+ cells? Any functionally related differentially expressed genes between the subtypes of fibroblasts contributing to tendon/ligament development.
-In addition to pro-inflammatory subtypes of fibroblasts, CD200+ DKK3+ fibroblasts have recently been described in adults. Functionally, these cells contribute to the resolution of inflammation. The authors should re-analyse their entire data set and also translate the developmental processes to this subtype of terminally differentiated fibroblasts and bring them in context to their hypotheses.

Reviewer #3

(Remarks to the Author)
The study by Davidson et al. addresses a long-standing and challenging question in rheumatology: why are proximal interphalangeal (PIP) joints more susceptible to inflammatory arthritis, while distal interphalangeal (DIP) joints are more prone to osteoarthritis. Using single-cell RNA sequencing (scRNAseq), high-resolution X-ray tomography, image analysis tools, and a combination of elegant molecular, spatial, and developmental analyses, the authors identify and characterize homeostatic synovial fibroblast populations that define the inflammatory and destructive layers of the synovium in fetal joints.

A key observation in the study is the differential enrichment of Pi16+ fibroblasts in fetal PIP joints compared to DIP joints. Specifically, the authors report 24.5% (± 7.0%) of Pi16+ fibroblasts in PIP joints versus 15.2% (± 6.3%) in DIP joints. They propose that this enrichment contributes to the differing susceptibilities of these joints to various types of arthritis in adulthood. Additional factors, such as differences in mechanical stress, structural complexities, proximity of Pi16 cells to endothelium and synovial volumes, are also acknowledged as potential contributors but are not further addressed.

Despite its intriguing findings, the study has significant weaknesses and limitations. The conclusions are primarily based on observational data, with no functional validation. The central hypothesis that the differential enrichment of Pi16+ fibroblasts underpins joint-specific susceptibility needs to be experimentally tested through studies in appropriate cellular or animal models. Moreover, further exploration is required to confirm these findings in other biological contexts where Pi16+ cells may influence inflammation susceptibility. Finally, the mechanisms by which Pi16+ fibroblasts contribute to joint inflammation and arthritis susceptibility remain poorly understood in the present work and need to be further investigated.

Decision Letter:

Our ref: NI-A38918A

23rd Mar 2026

Dear Dr. Buckley,

Thank you for submitting your revised manuscript "The embryonic origins of site-specific arthritis" (NI-A38918A). It has now been seen by the original referees and their comments are below. The reviewers find that the paper has improved in revision, and therefore we'll be happy in principle to publish it in Nature Immunology, pending minor revisions to satisfy the referees' final requests and to comply with our editorial and formatting guidelines.

We will now perform detailed checks on your paper and will send you a checklist detailing our editorial and formatting requirements in about a week. Please do not upload the final materials and make any revisions until you receive this additional information from us.

If you had not uploaded a Word file for the current version of the manuscript, we will need one before beginning the editing process; please email that to immunology@us.nature.com at your earliest convenience.

Thank you again for your interest in Nature Immunology Please do not hesitate to contact me if you have any questions.

Sincerely,

Stephanie Houston, PhD
Senior Editor
Nature Immunology

Version 1:

Reviewer comments:

Reviewer #1

(Remarks to the Author)
All questions are thoroughly addressed. Congratulations to the authors on a tremendous piece of work. I recommend publication in its revised form.

Reviewer #2

(Remarks to the Author)
The authors made a concerted effort to address reviewer comments that entailed significant experimental work with fibroblasts freshly isolated from embryonic joint tissues across three independent donors. While definitive mechanistic insights were not readily captured from these studies the authors did make important comparative observations regarding the responses of Pi16+ and Pi16- fibroblasts to inflammatory stimuli that will be of interest to the field.

Further, the authors searched for the presence of Pi16+ fibroblasts with similarity to embryonic joint-derived fibroblasts in other tissues from publicly available datasets and atlases. It would have been equally interesting and illuminating to search for signatures of the in vitro, inflammatory stimuli-treated Pi16+ cells in the adult tissue datasets and atlases. However, I will not delay publication of this manuscript by asking the authors to perform additional analyses.

This is a strong manuscript describing highly novel and fundamental findings that stand to advance the our understanding of joint and fibroblast biology during development, with relevance to pathobiology of joint diseases.

Open Access This Peer Review File is licensed under a Creative Commons Attribution 4.0 International License, which permits use, sharing, adaptation, distribution and reproduction in any medium or format, as long as you give appropriate credit to the original author(s) and the source, provide a link to the Creative Commons license, and indicate if changes were made.
In cases where reviewers are anonymous, credit should be given to 'Anonymous Referee' and the source.
The images or other third party material in this Peer Review File are included in the article’s Creative Commons license, unless indicated otherwise in a credit line to the material. If material is not included in the article’s Creative Commons license and your intended use is not permitted by statutory regulation or exceeds the permitted use, you will need to obtain permission directly from the copyright holder.
To view a copy of this license, visit https://creativecommons.org/licenses/by/4.0/
